# Effects of atrazine on the HPG and HPA axes and steroidogenic pathways in females: relevance to reproductive function and breast, ovarian and uterine cancer

**DOI:** 10.3389/ftox.2025.1686703

**Published:** 2026-01-05

**Authors:** Ralph L. Cooper, James W. Simpkins, Charles Breckenridge

**Affiliations:** 1 Quality Scientific Solutions, LLC, Waynesboro, VA, United States; 2 Department of Neuroscience, West Virginia University, Morgantown, WV, United States

**Keywords:** chlorotriazines, atrazine, mode of action, female, estrogen receptors, aromatase, signaling pathways, steroidogenesis

## Abstract

We reviewed the mode of action (MOA) underlying the effect of the chlorotriazines on female reproduction and mammary tumor development in rats. Age-associated changes in the HPO hormonal environment of the female drive the development of mammary gland tumors in several rat strains. The adverse outcome pathway for tumor development involves a disruption of the ovulatory surge of luteinizing hormone (LH) caused by changes in the hypothalamic control of LH release. The ensuing persistence of unruptured ovarian follicles produces elevated blood estradiol (E2) and prolactin, both known to induce mammary gland tumors. High doses of atrazine induce premature reproductive aging and elevated E2, which is commonly found later in aging female rats. The change in HPO in aging rodents is distinctly different from that seen in aging women. In humans, reproductive aging (menopause) is driven by the loss of ovarian follicles and ensuing low serum E2. Alternate MoAs were examined, including the effect of atrazine on estrogen synthesis, atrazine’s potential to bind to estrogen receptors, Erα, Erβ, or G-protein coupled, estrogen receptors (GPER) *in vitro*. The chlorotriazines do not bind to ER receptors; high doses may have anti-E2 effects. MOAs hypothesized from *in vitro* studies were of limited utility in predicting *in vivo* effects of atrazine because of the effects of metabolism and the kinetics of elimination *in vivo*. A review of the epidemiology literature indicated there is no consistent evidence of a causal association between chlorotriazine exposure and the incidence of breast, ovarian, or uterine cancers in women.

## 1 Introduction

**FIGURE 1:**
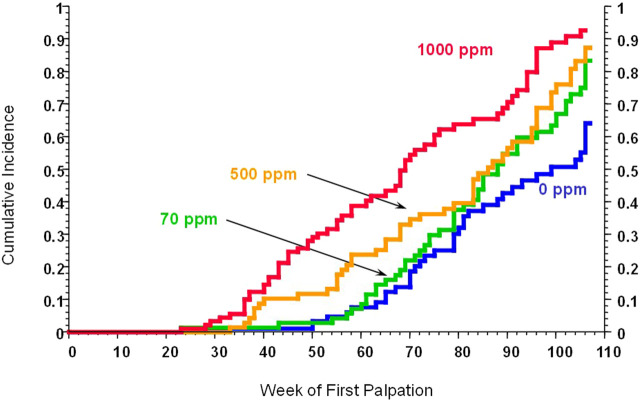
Kaplan-Meier Estimates of Mammary Tumor Incidence in Atrazine-Treated Female Sprague-Dawley Rats (Data from Mayhew, 1986, and summarized by Stevens et al., 1999 [SDR1]). Figure 1 is a plot of the cumulative incidence of mammary tumors in the control group (blue), the 70 ppm atrazine-treated group (green), the 500 ppm atrazine-treated group (orange), and the 1,000 ppm atrazine-treated group (red). Tumor masses were detected by weekly palpation of each animal until it was sacrificed. Tumor diagnoses (adenocarcinomas or fibroadenomas) were confirmed by microscopic examination by a board-certified pathologist. Statistical analyses of the cumulative tumor incidence in each group indicated that female SD rats in the 500 and 1,000 ppm atrazine-treated groups displayed a statistically significant earlier appearance of mammary tumors over the 104-week treatment period.

The effect of atrazine on the incidence and earlier appearance of mammary gland tumors in female Sprague-Dawley rats was first described in a study by Mayhew (1986) (Figure 1). This study triggered extensive research (Wetzel et al., 1994; Stevens et al., 1994; Simpkins et al., 1998; Eldridge et al., 1999b; Simpkins et al., 2011; Supplementary Table S1) aimed at understanding atrazine’s mode of action and the biological mechanisms underlying these effects. As the weight of evidence suggests that atrazine is neither genotoxic nor mutagenic (Brusick, 1994; Hauswirth and Wetzel, 1998; Jowa and Howd, 2011), we evaluated alternative mechanisms to explain the early onset of mammary gland tumors in laboratory rats. Towards this end, an exhaustive review was conducted examining the relevant literature on the effect of atrazine on the neuroendocrine control of ovarian function, estrogen synthesis, estrogen receptor signalling, and relevant signal transduction pathways potentially triggered by atrazine and its chlorometabolites, deethylatrazine (DEA), deisopropylatrazine (DIA), and diamino-chlorotriazine (DACT). The aim was to provide an updated perspective on the mode and mechanism of action of atrazine on reproductive functions and potential carcinogenicity.

For our literature search, we relied on an EndNote Library, which consisted of 39,537 references as of 13 May 2025. Initially, the library was constructed based on a 2008 PubMed search. The searches were all conducted using the term “atrazine.” There were no limitations on date, language, or other factors. Subsequently, the library was updated by searching the following databases: Agricola, CiNii, EMBASE, PASCAL, SciFinder, Toxline, and WorldCat. The library is currently kept up-to-date through weekly searches of the following databases: PubMed, BIOSIS Previews, and CAB Abstracts. Searches of the scientific literature for other topics (keywords, authors, etc.) related to the subject matter of this manuscript were conducted as needed by QS3 staff using Endnotes (PubMed) or Google Scholar.

We did not include a review of studies on the effect of atrazine on breast bud development because the results reported by Rayner et al. (2005) were not replicated by two independent laboratories (Davis et al., 2011; Hovey et al., 2011) subsequently. Likewise, we did not revisit the controversial “good estrogen-bad estrogen hypothesis” of Bradlow (Bradlow et al., 1995; Davis et al., 1993) because this topic has been exhaustively reviewed in the past (Safe, 1998). However, we conducted a comprehensive review to determine whether atrazine acts like an estrogen in biological systems.

Knowledge of the control of the ovarian cycle plays a critical role in understanding the effect of atrazine on the rodent reproductive system, and the appreciation of species-specific effects is critical to the age-dependent changes that occur in the uterus, breast, and ovary. Thus, we first provide an overview of the neuroendocrine control of ovulation in the rat with an emphasis on the impact of the chlorotrizines on the brain and pituitary control of the ovary. With advancing age, the neuroendocrine control of ovarian function is disrupted by the loss of the ovulatory surge of luteinizing hormone and the development of persistent ovarian follicles that continue to produce and secrete estradiol into the bloodstream (Huang et al., 1978), creating a hormonal environment conducive to mammary gland hyperplasia and tumor development. In contrast to the recognized changes in the neuroendocrine control of the LH surge in the rat (Cooper et al., 1986), the loss of ovarian cycles in women (menopause) is primarily driven by the progressive loss of follicles, both in quantity and quality (Finch et al., 1984; Wang et al., 2023).

In addition to evaluating the impact of atrazine on estrogen signaling, we assessed the role of the hypothalamic-pituitary-adrenal axis (HPA) in ovarian aging and other potential adverse health effects, as significant changes in the secretion of adrenal hormones have been identified.

Finally, several investigators have evaluated the effect of atrazine on signal transduction using various cell lines. The results of these studies have produced mixed outcomes as to the extent to which any of the observed molecular changes may contribute to adverse outcomes *in vivo* (including tumor development). The chlorotriazines, in general, continue to receive considerable attention in the scientific literature, and we trust that this review will inform the reader regarding atrazine’s effects and the doses required to produce such effects.

## 2 Regulation of the estrous cycle

**FIGURE 2:**
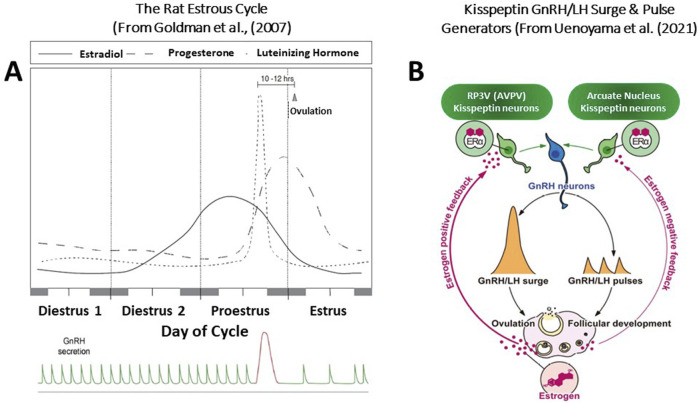
**(A)** The Rat Estrous Cycle and the GnRH/LH Surge and Pulse Generation. The timing and pattern of pituitary and ovarian hormone secretion are highly integrated, regulating follicular growth and maturation, ovulation, and the sexual behavior of the rat to maximize the probability that the ova will be fertilized. Any disruption of this hormonal pattern or disruption of the diurnal rhythm within the brain itself can have immediate and long-term adverse consequences. The rise in serum estradiol during the second day of diestrus and the morning of proestrus initiates the ovulatory surge of LH. This pattern of hormone secretion is strongly influenced by the daylight cycle. Over the cycle, the GnRH pulse generator controls the pattern of gonadotropin release, favoring FSH secretion during the follicular phase, maintaining follicular maturation. The rise in progesterone after ovulation results in a decrease in the LH pulse frequency. The highly regulated control of the ovarian cycle deteriorates with advancing age, leading to changes in the pattern of hormone secretion that contribute to the development of reproductive pathology, including mammary gland tumors. The well-defined LH surge occurs on the afternoon of proestrus, triggering ovulation and, along with prolactin (not shown), initiates luteinization of the granulosa cells within the follicle and the formation of the corpora lutea. Ovulation occurs approximately ten to 12 h after the LH surge, whereas sexual receptivity peaks during the intervening hours, maximizing the chances that fertilization will occur. **(B)** Kisspeptin-Mediated GnRH/LH Surge and Pulse Generators. Central mechanisms underlying the negative and positive feedback actions of estrogen on pulsatile and surge modes of gonadotropin-releasing hormone (GnRH)/luteinizing hormone (LH) release in female rodents. Estrogen production, along with follicular development, is stimulated by GnRH/gonadotropin pulses. During the follicular development period, low levels of circulating estrogen fine-tune GnRH/H pulses via the negative feedback action of estrogen. The estrogen negative feedback action is considered to be mediated by estrogen receptor α (ERα)-expressing kisspeptin neurons located in the arcuate nucleus (ARC). Estrogen production and release gradually increase along with the follicular development, and consequent high levels of circulating estrogen derived from mature follicles, in turn, induce GnRH/LH surge and hence ovulation via the positive feedback action of estrogen. The estrogen positive feedback action is likely mediated by ERα -ERα-expressing kisspeptin neurons located in the arcuate nucleus.

The female rat is a spontaneous ovulator. After puberty, female rats exhibit rhythmic four-to five-day estrous cycles. These cycles can be divided into three separate segments: a period of diestrus (lasting 2 to 3 days), proestrus (1 day), and estrus (1 day). Each segment is readily identified by observing the changes in vaginal cytology, which occur in response to fluctuations in ovarian steroid levels in the blood (Cooper et al., 1986; Goldman et al., 2007; Figure 2).

The key event of the estrous cycle is the occurrence of the ovulatory surge of luteinizing hormone (LH) from the pituitary. In the intact female rat, housed under controlled daily lighting conditions of 10 h of dark and 14 h of light, plasma LH remains low except for a brief period late in the afternoon on the day of vaginal proestrus. At this time, the serum LH concentration rapidly increases by approximately ten-to 20-fold (Cooper et al., 1980). Ovulation occurs approximately 10 to 12 h after this surge, whereas sexual receptivity peaks during the intervening hours, maximizing the chances that fertilization will occur.

The sequence of neuroendocrine events surrounding the ovulatory surge of LH includes both positive and negative feedback effects of the ovarian steroids on the brain and pituitary. Estradiol levels in the blood are relatively low on the day of diestrus I (Figure 2), begin to rise on the afternoon of the second day of diestrus, and reach their highest levels around noon on the day of proestrus. Serum progesterone concentration begins to rise on the morning of proestrus and reaches peak values later in the evening. The sequential pattern of rising estrogen levels followed by increasing progesterone levels has a synergistic effect on the brain and pituitary, serving to control the amount and timing of LH release (Mittelman-Smith et al., 2017; Toufaily et al., 2020).

### 2.1 GnRH-LH surge

**FIGURE 3:**
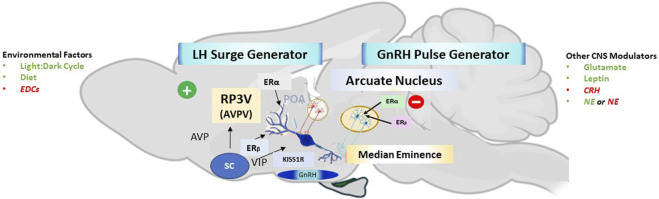
Hypothalamic Neuronal Circuitry Responsible for Pulsatile GnRH-LH Secretion and the GnRH-LH. Kisspeptin neurons in the arcuate nucleus (light blue neurons within a yellow circle) and the rostral periventricular area of the third ventricle (RP3V), which includes the anteroventral periventricular nucleus (AVPV) (red neurons within a white circle), play two distinct roles critical in regulating the ovarian cycle. ARC Kisspeptin neurons exhibit episodic activity that induces pulsatile GnRH and LH secretion through estradiol (E2) mediated negative feedback. These neurons are known as the ‘GnRH pulse generator’ (depicted on the right side of the figure). Synchronization of these neurons is driven by NKB and dynorphin; NKB stimulates, whereas dynorphin inhibits kisspeptin release (not shown). Ovarian steroid hormones influence the pulse frequency along with peripheral factors [glutamate (stimulatory), leptin (stimulatory), CRH (inhibitory), Norepinephrine (stimulatory {green} or inhibitory {red}] which, in turn, maintain the proper concentration of FSH and LH in the blood during the follicular phase and luteal phase of the cycle. By contrast, the RP3V Kisspeptin-neurons induce the mid-cycle, ovulatory LH surge through E2-mediated positive-feedback (depicted on the left side of the figure). The estrogen receptor (ERα) is responsible for mediating both negative and positive feedback from E2. ERα can signal either by translocation to the nucleus and recruitment of cofactors to the estrogen response element (ERE) (classical pathway), or by recruitment of other transcription factors not via the ERE (non-classical pathway). Notably, E2-induced positive feedback occurs via the classical pathway, whereas negative feedback is mediated via the non-classical pathway. Estrogen receptor β (ERβ) has been shown to potentiate E2 positive feedback in the RP3V in the presence of high E2 levels and has an inhibitory effect on ERα in the AVPV in the presence of low E2 levels. Regardless of E2 levels, the ratio of ERα:ER*β* is twice as high in the ARC as in the AVPV. ARC, arcuate nucleus; AVPV, anteroventral periventricular nucleus; CRH, corticotropin-releasing hormone; EDCs (Endocrine Disruptor Compounds) E2, estradiol; ERα, estrogen receptor α; ER*β*, estrogen receptor*β*; ERE, estrogen response element; FSH, follicle-stimulating hormone; GnRH, gonadotropin-releasing hormone; KISS1R, kisspeptin receptor; LH, luteinizing hormone; NKB, neurokinin B; NE, Norepinephrine; RP3V, rostral periventricular area of the third ventricle. (From Herbison, 2020; Goodman et al., 2022; Stevenson et al., 2022).

The immediate stimulus for the ovulatory surge of LH is the enhanced liberation of gonadotropin-releasing hormone (GnRH) from the brain into the capillary network that bathes the pituitary (hypothalamic-hypophyseal portal system). Gonadotropin-releasing hormone is synthesized in neuronal cell bodies located in the rostral hypothalamus [preoptic area (POA)]. It is transported along axons projecting to the medial basal hypothalamus (median eminence area), where the terminals contact the capillary vessels of the hypothalamic-hypophyseal portal system. GnRH neurons do not contain estrogen receptors. Kisspeptin neurons in the rostral periventricular area of the third ventricle (RP3V) play a key role in relaying the positive feedback effects of estradiol to activate gonadotropin-releasing hormone (GnRH) neurons and drive a surge in the GnRH/luteinizing hormone level (Uenoyama et al., 2021; Goodman et al., 2022). During the “GnRH surge”, GnRH neuronal activity becomes highly synchronized under the influence of positive feedback from ovarian estradiol (Figures 2B, 3). This “estradiol signal,” which arises from the developing follicles in the ovary, activates kisspeptin neurons located in the anteroventral periventricular nucleus (AVPV) near the third ventricle labeled RP3V. This group of kisspeptin neurons is activated by estradiol via ERα receptors, leading to the synchronization of GnRH neuronal activity through the peptide hormone kisspeptin binding to the G-protein-coupled receptor, GPR-54, located on GnRH neurons (Smith et al., 2006; Uenoyama et al., 2021).

### 2.2 GnRH pulse generator

In rodents, a second set of neurons regulates the secretion of GnRH over the estrous cycle. The GnRH pulse generator, located in the arcuate nucleus near the median eminence, is comprised of a group of “KNDy” neurons that express the neuropeptides kisspeptin, neurokinin B, and dynorphin, respectively (See Figures 2B, 3). KNDy neurons are activated synchronously in an episodic manner, and these synchronized episodes consistently precede LH pulses (Moore et al., 2022; Stevenson et al., 2022). Episodic pulses of the GnRH peptide are released into the pituitary portal circulation, where they activate the pituitary gland to release FSH and LH during the follicular, ovulatory, and luteal phases of the estrous cycle. In the follicular phase, low estradiol levels are associated with a pulse frequency that is greater than that during the luteal phase. The high levels of progesterone after ovulation are responsible for the lower frequency of the pulses while the corpus luteum persists.

The secretion of prolactin, which is essential to corpora lutea formation, gestation, and lactation, is also under monoaminergic (particularly dopaminergic) regulation by the central nervous system (CNS). As with gonadotropin secretion, the timing and amount of prolactin release are influenced by environmental factors and steroid hormones.

Input to the GnRH/LH surge mechanism is also derived from daily signals that originate in the suprachiasmatic nucleus (SCN). This signal appears to be carried in part by arginine vasopressin (AVP)-positive afferents from the SCN to the ERα expressing kisspeptin cells in the RP3V and by SCN projections that contain vasoactive intestinal peptide (VIP to GnRH cell bodies in the POA (Goodman et al., 2022). Thus, the core neuronal elements of the GnRH and LH surge generator mechanism in rats and mice consist of VIP and AVP neurons in the SCN, as well as estrogen-responsive RP3V kisspeptin neurons, and POA GnRH soma and processes that project to the median eminence.

Differences between the mechanisms controlling ovulation in primates and human females have been reviewed in detail elsewhere (Plant, 2012) and will not be discussed here.

### 2.3 Reproductive senescence in the rat

There is general agreement that reproductive aging in the untreated female rats is brought about through a disruption of the luteinizing hormone (LH) surge generator in response to prolonged exposure to endogenous estrogens, primarily ovarian estradiol (Schipper et al., 1981; Brawer et al., 1986; Hung et al., 2003; Finch et al., 1984). In brief, these investigators have demonstrated that the elevated serum estradiol concentrations present in the young-adult female, during the estrous cycle (particularly vaginal proestrus), lead to histopathological changes in the hypothalamic neuronal circuit that controls the secretion of LH (Finch et al., 1984). Between 8 and 12 months of age (depending on the strain and other environmental factors), cumulative damage approaches the critical point where a sufficient amount of LH to induce ovulation is no longer present. Before this point, in the still, regularly-cycling female, there are noticeable shifts in the timing and the amplitude of the surge (Cooper et al., 1980; Wise et al., 1990), indicating that changes within the hypothalamic-pituitary control of ovulation are beginning. It has been demonstrated that the amount of prior estrogen exposure in females will also modify the age at which these changes in the regulation of the LH surge will occur. For example, exposing females to estrogens in adulthood or neonatally (Handa and Gorski, 1985; Handa et al., 1985) can bring about a premature disruption of regular ovarian cycles that are preceded by the same pattern of change in LH secretion observed in the untreated middle-aged female. These findings underscore the importance of the internal endocrine milieu in pacing the disruption of the hypothalamic control of the LH surge.

According to Meites (1972), “*The two most important hormones for development and growth of mammary tumors in rats are prolactin and estrogen, both under control of the pituitary gland and the brain. Estrogen stimulates prolactin secretion and acts with prolactin directly on the mammary tissues to promote tumorigenesis.”* With advancing age, the regular pattern of the ovarian cycle present in young adults typically gives way to a pattern of persistent estrus. This is because the LH surge in most rat strains, including the middle-aged Sprague-Dawley female, will not be sufficient to induce ovulation. As ovulation fails in the Sprague Dawley rat, the ovaries contain numerous well-developed follicles and no corpora lutea (Huang and Meites, 1975).

Additionally, the persistent estrus female exhibits a daily diurnal prolactin surge immediately preceding the dark period (Damassa et al., 1980). This contrasts with the prolactin peak that occurs only on the day of vaginal proestrus (i.e., every 4 or 5 days) in the young-regularly cycling female. It is this combination of estradiol and daily prolactin surges that promotes the development of mammary gland tumors (Meites, 1972; Ciocca et al., 1982; Russo, 2015). As female rats continue to age, some may exhibit a pattern of repetitive pseudopregnancy, characterized by the formation of corpora lutea and the secretion of appreciable concentrations of progesterone. These females also have a daily prolactin peak, but it occurs at the end of the dark period (Damassa et al., 1980).

The changes described above in ovarian function of the female Sprague Dawley (SD) rat are different from those observed in female Fischer-344 (F-344) rats. F-344 rats continue to cycle until later in life (∼15 months) than SD females (∼10–12 months), and when regular cycles are interrupted, they assume a pattern of repetitive pseudopregnancies, not persistent estrus. In the Fischer-344 females, the vaginal smear is primarily leukocytic (indicative of low estradiol), and the ovaries contain many well-developed corpora lutea (Huang and Meites, 1975). Serum estradiol levels remain low, and progesterone concentrations are elevated, and presumably, the daily prolactin surge occurs towards the end of the dark period (Huang et al., 1978; Damassa et al., 1980). As it is well-documented that mammary tumors in the rat are hormonally dependent (Ciocca et al., 1982; Russo, 2015; Russo and Russo, 1996), the stark difference in the post-reproductive endocrine milieu in unexposed SD versus Fischer-344 females is responsible for the development of mammary gland tumors in SD females and not in the F-344. This strain difference in the pattern of reproductive aging is essential for understanding the relevance of the post-reproductive endocrine environment and mammary gland tumors, as atrazine does not induce early reproductive senescence or the early appearance of mammary gland tumors in the Fischer-344 female.

### 2.4 Reproductive aging in humans

In women, age-related changes in ovarian function typically begin in the mid-30s, characterized by a gradual decline in the number of ovarian follicles available for ovulation, which is accompanied by a decrease in fertility. During this period, known as the menopause transition, a compensatory hormonal adjustment occurs in the hypothalamic-pituitary-gonadal (HPG) axis, which helps maintain follicular development and estrogen secretion despite the declining pool of ovarian follicles. It is generally accepted that circulating FSH levels begin to rise for many years during the menopause transition as a result of declining ovarian inhibin secretion that reflects decreasing numbers of ovarian follicles and a decreasing number of granulosa cells within the follicles that remain. Rising FSH levels increase aromatase activity in the granulosa cells. In women who continue to cycle, FSH secretion helps to maintain or elevate estradiol levels during the early follicular phase. Increased FSH is also associated with an acceleration of the maturation of the dominant follicle and results in a decrease in the length of the follicular phase. Despite the decrease in the follicular phase, regular cycles within the normal range of 25–35 days can be maintained. The duration of the luteal phase is unchanged in older women who continue to cycle, in contrast to reproductive aging in rodents (Simpkins et al., 2011; Supplementary Table S2). However, plasma progesterone levels are lower than in “young” women (around 20–37 years of age), and estradiol levels are marginally higher. The menopause transition is characterized by marked variability in follicle development, ovulation, bleeding patterns, and symptoms of hyper- and hypoestrogenism. Menopause onset, which occurs clinically with the final menstrual period (FMP), is characterized by the absence of ovarian estradiol (E2) secretion (Hall, 2015).

## 3 Atrazine increased mammary tumor incidence and decreased time-to-tumor onset in SD rats

Mayhew (1986) reported a dose-related, statistically significant incidence of mammary tumors in female SD rats fed atrazine in the diet at levels of 500 or 1,000 ppm for 104 weeks (Figure 1; See Study SDR1 in Stevens et al., 1999). An earlier appearance of mammary tumors was also evident in the two high-dose groups (500 and 1,000 ppm). The carcinogenic no-observed-effect level (NOEL) in this study was 10 ppm. The atrazine dose received in the 10 ppm dietary group ranged from 1.4 to 0.7 mg/kg/day during the first 13 weeks of the study and from 0.6 to 0.4 mg/kg/day from month 4 to 24.

A second carcinogenicity study was conducted in ovariectomized female SD rats (See study SDR4 in Stevens et al., 1999) at dietary levels of 0, 25, 50, 70, or 400 ppm. The incidence of mammary tumors in this study was compared to that of intact SD rats fed atrazine in the diet at the same levels (See study SDR5 in Stevens et al., 1999). The results indicate that atrazine had no effect on mammary tumor incidence in ovariectomized females (i.e., zero mammary tumor incidence in all control and atrazine-treated groups). In contrast, intact female SD rats displayed non-monotonic, statistically significant elevations in the incidence of mammary tumors (fibroadenomas and adenocarcinomas) at atrazine feeding levels of 50 ppm or higher. The early onset of mammary tumors only occurred in the 400 ppm, atrazine-treated group.

**FIGURE 4:**
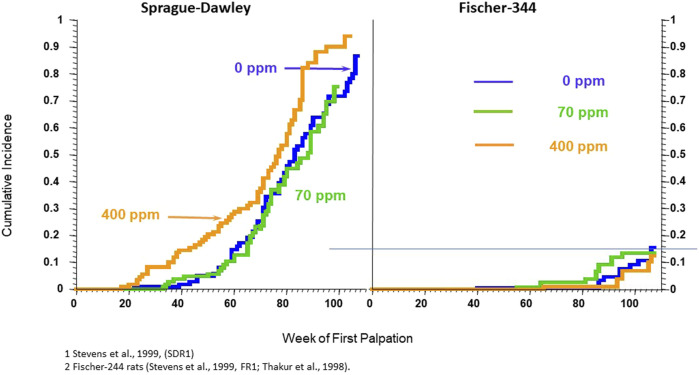
Cumulative percent incidence of mammary tumors in female Sprague-Dawley (Left Panel) and Fischer-344 (Right Panel). SD and F-244 rats werefed atrazine in the diet at concentrations of 0 (blue), 70 (green), 500 (tan), or 1,000 (red) ppm for 24 months beginning at 6–7 weeks of age on week zero and ending at 104 weeks. Tumors were proven at necropsy. 60 animals/group. (From: Stevens et al., 1999; Study SRD1 – Left Panel). and Stevens et al., 1999, Study FR1 and Thakur et al., 1998). See the legend for Figure 1 for details on the Kaplan-Meier plots. In this study, the 400-ppm atrazine-treated female SD rats displayed a statistically significant earlier appearance of mammary tumors; there were no effects of atrazine on the cumulative incidence of mammary tumors in female Fischer-344 rats in either the 70 or 400 ppm atrazine-treated groups. The incidence of mammary tumors in untreated control Fischer-344 rats was almost 6-fold lower than in untreated female SD rats.

In a third investigation, the effect of atrazine on the incidence of mammary and pituitary tumors in female SD rats fed diets containing 0, 70, or 400 ppm for 24 months (See Study 3 and 4 combined in Wetzel et al., 1994) were compared to the results from female F-344 rats fed atrazine at levels of 0, 10, 70, 200 or 400 ppm (See study 1 and 2 combined in Wetzel et al., 1994). Female SD rats in the high-dose group (400 ppm) displayed a statistically elevated incidence of mammary tumors (Figure 4, Left Panel) and pituitary tumors during the period from zero to 52 weeks of treatment, but not from zero to 104 weeks (Data not shown). The carcinogenic NOEL in this study was 70 ppm (doses ranged from 6.97 during Week 1 to 2.97 mg/kg/day during Week 104; Thakur, 1992, Hazleton Study No. 483-275). Atrazine did not affect mammary tumor incidence in female F-344 rats at any dose (Figure 4, Right Panel; data for only the 0, 70, and 400 ppm groups shown), although pituitary tumor incidence was slightly reduced in the high-dose group from week 1 to 52 weeks of treatment, but not from week 1 to 104 weeks.

Stevens et al. (2001) published a structure-activity relationship (SAR) analysis of rodent carcinogenicity studies conducted on four classes of triazine herbicides: chlorotriazines, s-methyl-substituted triazines, O-methyl-substituted triazines, and asymmetrical triazines. Carcinogenicity studies conducted in rats indicated that the incidence of mammary tumors was observed in high-dose groups of animals exposed to atrazine, simazine, propazine, terbuthylazine, cyanazine, and terbutryn. Hydroxyatrazine, which is not a chloro-s-triazine but rather a plant metabolite of atrazine, was negative in the rodent carcinogenicity bioassays. The carcinogenic potential of atrazine chloro-metabolites (deethylatrazine [DEA], deisopropylatrazine [DIA], and diaminochlorotriazine [DACT]) has not been evaluated in lifetime studies. None of the chlorotriazines caused mammary tumors in mice.

### 3.1 Atrazine accelerates age-related changes in the estrous cycle of sprague- dawley rats

**FIGURE 5:**
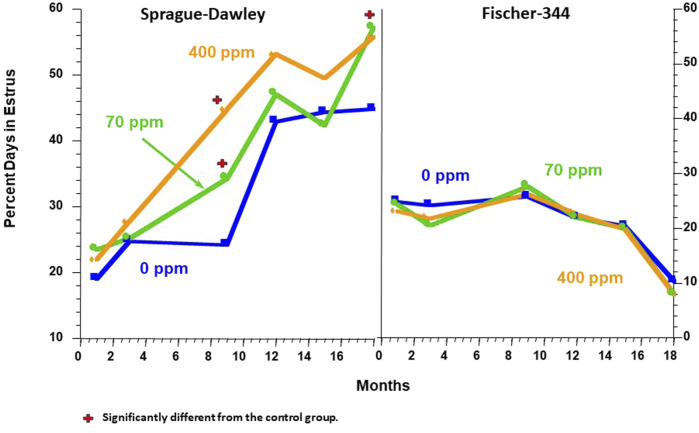
Comparison of the Effect of Atrazine on the Estrous Cycle in the Female Sprague Dawley (Left Panel) vs. the Fischer-344 Rat (Right Panel). Data from Table 2 of Wetzel et al. (1994). In these studies, all survivors out of 10 rats/sex/group assigned to serial sacrifice subgroups were killed after months 1, 3, 9, 12, 15, 18, or 24 months of treatment. Two weeks prior to sacrifice, daily vaginal lavages were collected on glass microscopic slides, air dried, and examined microscopically by Dr. Eldridge. The slide was classified as being in one of three stages of the estrous cycle (See Figure 2A). The percentage of days that each animal was in estrus was determined, and the group mean data over each observation period were plotted. The results indicated that female SD rats fed atrazine in the diet at 70 and 400 ppm spent significantly more days in estrus than did the controls at 9 and 18 months. There was no effect of atrazine treatment on the percentage of days in estrus in Fischer-344 females.

Wetzel et al. (1994) observed that female SD rats administered 400 ppm of atrazine in the diet advanced the age-related changes in the estrous cycle, which generally commenced in untreated female SD rats around 12 months of age (Figure 5, left panel). In contrast, the 400-ppm atrazine-treated females Figure 5) displayed a clear, early pattern of persistent estrous episodes. The percentage of days spent in estrus in the 400-ppm atrazine-treated group increased from ∼25% at 3 months to 45% at 9 months, reaching a plateau of 55% by 12 months (Figure 5, Left panel). The results for SD rats were confirmed in a second study by Eldridge et al. (1999a), who examined the ovarian cycle of individual rats from the time they were exposed to dietary atrazine (0, 25, 50, and 400 ppm/day) beginning at 7–8 weeks of age and maintained on the diet for 6 months. By the end of 26 weeks, less than 50% of vaginal smears for controls were estrus smears (Supplementary Figure S1), whereas in the 400-ppm atrazine-treated SD rats, ∼65% of the vaginal smears were estrus smears (Supplementary Figure S2). Collectively, these findings support the hypothesis that atrazine causes earlier reproductive aging in female SD rats. In contrast, no effect of 400 ppm atrazine on the estrous cycle was observed in F-344 rats (Figure 5, right panel).

In summary, the prolongation of the number of days that 400 ppm, atrazine-treated SD females spent in estrus compared to controls was likely due to multiple failed ovulatory attempts resulting in an increased number of days that SD females were exposed to endogenous estrogen secreted by ovarian follicles and the number of recurrent prolactin surges that were coupled to recurring LH surges. These early changes in the estrous cycle of atrazine-treated SD rats resulted in an earlier appearance of mammary tumors. Ovariectomized female SD rats treated with atrazine did not develop any mammary tumors (See study SDR4 in Stevens et al., 1999), indicating that atrazine had no direct effect on the mammary gland of SD rats. Finally, there were no effects of atrazine treatment on the estrous cycle or mammary tumor incidence in female Fischer-344 rats.

The elevated incidence of mammary gland adenocarcinomas in SD rats was best predicted by the number of days the animals spent in estrus (Supplementary Figure S3), while an elevated incidence of mammary gland fibroadenomas correlated with the presence of mammary gland acinar lobular development and prolactin-induced galactoceles found at necropsy, with a greater prevalence in atrazine-treated SD rats (Supplementary Figure S4). The prolactin result is consistent with the observation that there is a prolactin surge concomitant with the LH surge. Failure to achieve the LH ovulatory threshold in atrazine high-dose females late in proestrus suggests that the mammary gland in atrazine-induced acyclic females would be exposed to multiple days of elevated prolactin.

**FIGURE 6:**
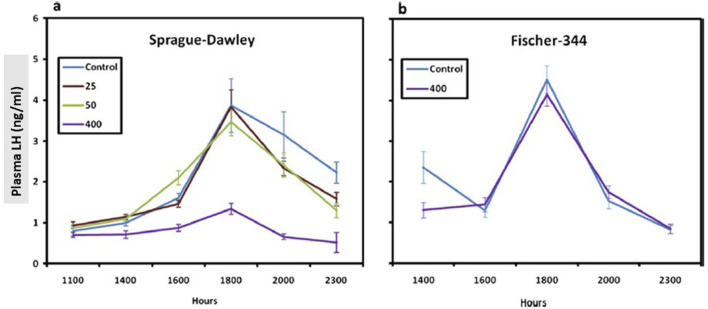
Comparison of the Normalized^1^ LH Surge in the Sprague-Dawley **(a)** to the Fischer-344 Rat **(b)** after 6 months of Atrazine Treatment (From Simpkins et al., 2011). Female SD rats were administered atrazine in the diet for 6 months at concentrations of 0, 25, 50, or 400 ppm; Two groups of female Fischer-344 rats were administered 0 or 400 ppm atrazine in the diet. In both studies, six subgroups of 10–15 rats/sex/group were designated for LH surge evaluation, and animals were ovariectomized under isoflurane inhalation anesthetic. Six days after ovariectomy, estradiol (4 mg/mL in sesame oil) was administered via a subcutaneously implanted 8 mm capsule. Three days later, commencing 8 h after room lights were turned on (0500 h) and animals were serially sacrificed at 1,100, 1,400, 1,600,1,800, 2,000, or 2,300 h biological time (Lights were turned off at 1,900 h). Plasma LH concentration levels were evaluated by radioimmunoassay (RIA), and group means and standard deviations were plotted. Overall, the results indicate that 400 ppm atrazine suppressed the LH surge in Sprague-Dawley but not Fischer-344 females.

**FIGURE 7:**
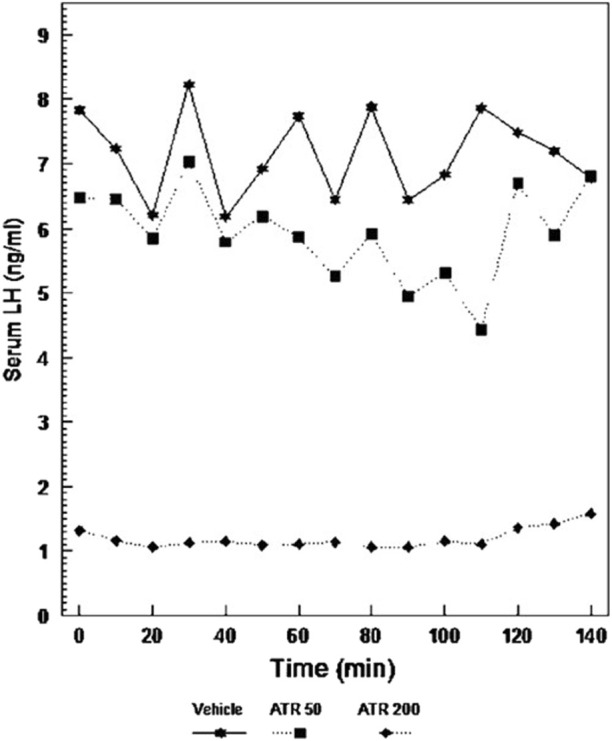
Pulsatile-plasma LH levels in Long Evans rats given atrazine. Adult females were ovariectomized for at least 4 weeks prior to testing (From Cooper et al., 2007). Pulsatile-plasma LH levels in Long Evans rats given Atrazine i.p. at doses of 0, 50, or 200 mg/kg/day for 3 days, the 200 mg dose caused a significant suppression of LH release. Although the pulse pattern in the 50 mg/kg dose was disorganized, there was no difference in the overall secretion of this hormone.

In a 6-month feeding study conducted in SD rats, a statistically significant reduction in the LH surge was observed in the 400-ppm atrazine-treated females after 6 months of treatment (Figures 6, 7, left panel). The no-observed-adverse-effect level (NOAEL) was 50 ppm (3.65 mg/kg/day). There was no effect of atrazine on LH at the highest dose administered (400 ppm) to F-344 rats for 6 months (Figure 7; right panel).

### 3.2 Atrazine reduces the LH surge by altering pulsatile GnRH release from the hypothalamus

The interruption of ovarian cycling in middle-aged female SD rats suggests that exposure to atrazine may lead to early reproductive senescence by altering the hypothalamic control of GnRH. This conclusion was supported by the observation that atrazine (100 ƞM) did not alter baseline or GnRH (85 nM)-stimulated LH release from the pituitary examined using a perifusion procedure. In the same experiment, atrazine did not alter baseline or thyrotropin-releasing hormone (TRH) (100 mM)-stimulated prolactin release (Cooper et al., 2000). Furthermore, when the anterior pituitary tissue was auto-transplanted beneath the kidney capsule, where it was no longer under the inhibitory control of hypothalamic peptides, atrazine did not alter the serum concentration of prolactin. Further support for the conclusion that the hypothalamus was the target site for the effect of atrazine on the LH surge, Cooper et al. (2000) showed that three daily oral doses of 300 mg/kg atrazine administered to intact female Long Evans (LE) rats nearly completely suppressed the LH surge (Figure 15). In atrazine-treated females, the intravenous administration of 50 ng of GnRH through a cardiac catheter at time zero restored the LH surge to levels seen in untreated control LE rats.

Female LE rats displayed significantly decreased LH and prolactin surges after 3 days of atrazine administration at oral gavage doses ≥ 100 mg/kg/day. At a dose of 50 mg/kg/day, the LH and prolactin surges had recovered to control levels by 6 h. In contrast, there was no effect of atrazine on the LH or prolactin surge in SD females administered ≤ 200 mg/kg/day atrazine for 3 days. The 300 mg/kg/day atrazine group of SD rats displayed a reduced LH response at 6 h and significantly reduced prolactin surge at 0, 1, 3, and 6 h. Both the prolactin and LH surges were significantly reduced in SD rats after 21 days of treatment. In addition, the onset of the estrogen-induced LH and prolactin surges in the LE females was delayed in the atrazine-treated animals compared to controls (Cooper et al., 2000). The delay in the onset of the surge is similar to that observed in middle-aged females before the age-associated disruption of ovarian cycles (Cooper et al., 1980).

Simpkins et al. (1998) reported that a high dose of atrazine administered by gavage in ovariectomized, estrogen-primed female SD rats delayed the onset and suppressed the amplitude of both the LH and prolactin surges. McMullin et al. (2004), who administered atrazine or DACT to ovariectomized, estrogen-plus progesterone-primed SD females, confirmed the effect of atrazine and DACT on LH surge. Minnema et al. (2001a); Minnema, 2001b; Minnema (2002) reported that the administration of equimolar concentrations of atrazine (400 ppm), simazine (347 ppm), or DACT (274 ppm) to intact female SD rats for 6 months suppressed the LH surge. The highest dose tested for atrazine by Minnema et al. was the same as that used in an oncogenicity study, where early reproductive senescence and a mammary gland tumor response had been characterized in the SD females.

**FIGURE 8:**
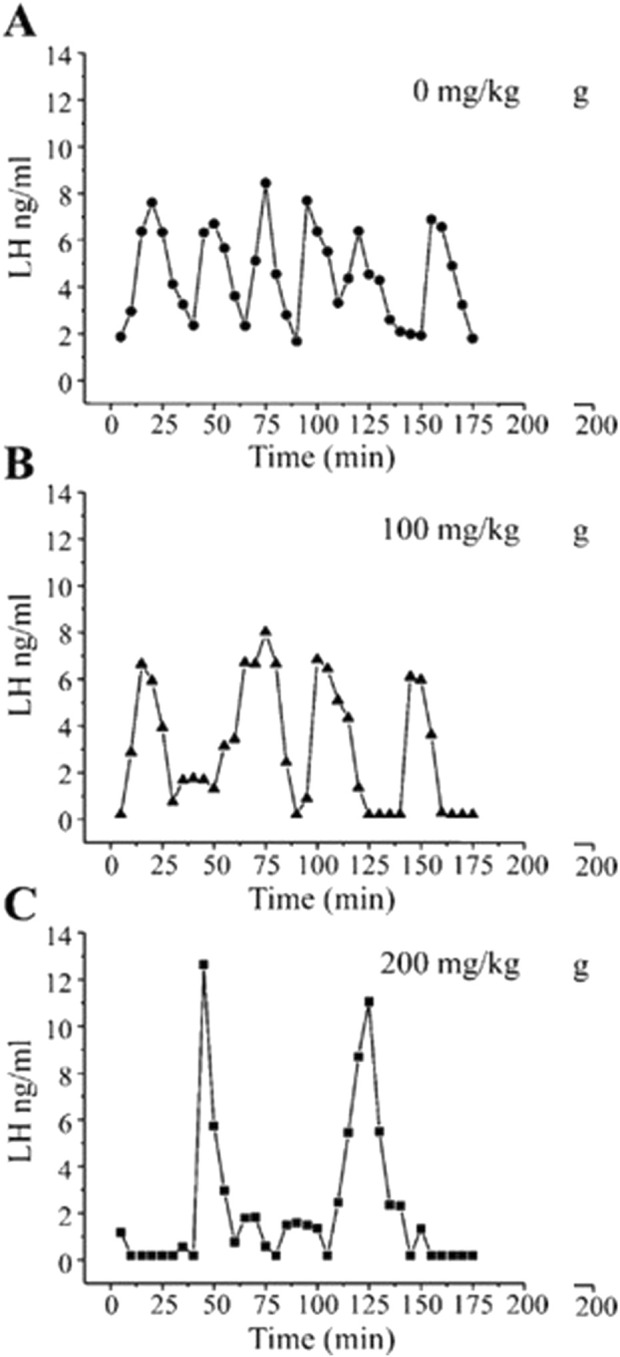
Pulsatile plasma LH levels in Wistar rats given atrazine by gavage at doses of 0, 100, or 200 mg/kg/day for 4 days (From Foradori et al., 2009). Young adult female Wistar rats were single-housed with a 12-h light: dark cycle with *ad libitum* access to food and water. After acclimatization, all animals were ovariectomized under anesthesia, followed by a 7 to 8 days recovery period. A jugular cannula was implanted to permit serial blood collection from atrazine-treated rats (0, 50, 100, or 200 mg/kg/day administered by gavage for 4 days). Three to 4 hours after the last atrazine dose, 150 µL of blood was collected repeatedly over a 3-h period and analyzed for LH by radioimmunoassay. The results indicated that atrazine at a dose of 100 mg/kg/day decreased pulse frequency and at 200 mg/kg/day decreased LH pulse frequency and increased pulse amplitude.

Cooper et al. (2007) demonstrated that GnRH pulses were significantly suppressed in long-term ovariectomized LE rats following daily intraperitoneal (i.p.) injections of atrazine at a dose of 200 mg/kg/day. The LH pulse pattern was altered, and amplitudes were reduced in the 50 mg/kg group, although these changes were not significantly different from those in untreated controls (Figure 8A). Foradori et al. (2009) confirmed these findings in ovariectomized Wistar rats administered atrazine by daily gavage at doses of 0, 100, or 200 mg/kg/day. Blood samples were collected every 5 min from indwelling catheters during the 200-min collection period. Controls displayed seven LH pulses during this interval of time with a baseline of 2 ng/mL and peaks of approximately 7–8.5 ng/mL (Figure 8B). The 100 mg/kg atrazine dose group had a baseline LH level near zero, with only four LH pulses, each with LH levels ranging from 7 to 8 ng/mL. The 300 mg/kg atrazine-treated group also had a zero LH baseline but displayed only two LH pulses whose amplitudes were approximately 12–13 ng/mL. These results suggest that GnRH accumulates between pulses, and when pulse frequency declines, pulse amplitude increases proportionally.

Kimura et al. (2019) examined the effect of five daily doses of 0, 100, or 200 mg/kg/day atrazine on kisspeptin regulation of GnRH release in ovariectomized, estrogen-primed Jcl: Wistar rats. Atrazine, at doses ≥ 100 mg/kg/day, significantly reduced the LH surge (peak level and AUC) but did not alter baseline LH levels or decrease hypothalamic GnRH levels at the highest dose tested (100 mg/kg/day).

Kimura et al. (2019) also administered a single subcutaneous dose of kisspeptin (0, 0.01, 0.1, and 10 nmol/kg) to ovariectomized, estrogen-primed rats that had received five daily doses of atrazine (100 mg/kg/day). The high dose of kisspeptin stimulated a larger LH surge in the 100 mg/kg/day atrazine-treated group compared to the untreated vehicle controls. These results suggest that GnRH neurons in atrazine-treated rats can be driven to produce an LH surge, presumably mediated by neuronal activity in kisspeptin neurons located in the arcuate nucleus and/or in the anteroventral periventricular nucleus (AVPN) of the hypothalamus.

Zorrilla et al. (2009) described the effect of atrazine, or DACT, on GnRH release from medial basal hypothalamic (MBH) explants maintained in a perifusion chamber. They found that baseline GnRH levels, pulse frequency, and pulse amplitude were reduced after 24 h of exposure to 10 or 100 µM atrazine or 100 µM DACT. In a second experiment, immortalized mouse hypothalamic GT7-1 cells exposed for 24 h to 100 µM atrazine or DACT also resulted in decreased GnRH baseline release and pulse frequency. The relevance of these findings, however, is difficult to assess given that 100 µM concentrations of atrazine and DACT are unlikely to be found *in vivo.*

Overall, the results from the above studies suggest that the effect of chlorotriazines on the LH surge may be mediated through a hypothalamic site of action involved in GnRH pulse and surge generation (Figures 2, 3). The effect of atrazine on the onset and peak amplitude of the LH surge, which is likely mediated by changes in GnRH output, as influenced by kisspeptin neurons in the arcuate nucleus and RP3V, is qualitatively similar to that observed in middle-aged female rats before the onset of reproductive senescence (Cooper et al., 1980). Iwata et al. (2019) suggested that the reduction of kisspeptin expression in neurons located in the arcuate nucleus of older female rats was linked to the reduction in plasma LH levels. The exact locus of atrazine’s effect on GnRH pulse and surge generation and the molecular target(s) remain to be elucidated.

## 4 Are the effects of the chlorotrazines on the HPG axis secondary to effects on the HPA axis?

**FIGURE 9:**
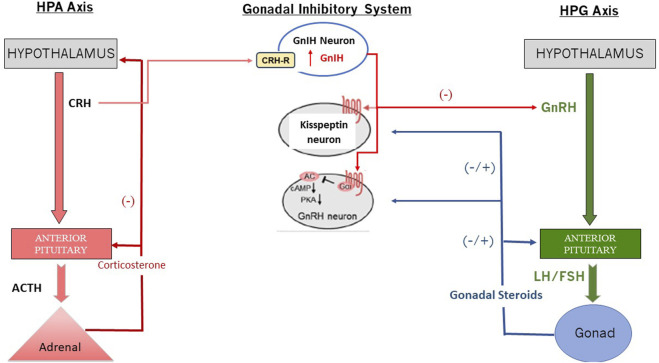
Interaction between the HPA and the HPG Axis via GnIH (Redrawn from Son et al., 2025). Abbreviations: cAMP, cyclic adenosine monophosphate; CRH: Corticotropin-Releasing Hormone; CRH-R: Corticotropin Releasing Hormone Receptor; FSH: Follicle Stimulating Hormone; GnIH: Gonadotropin Inhibiting Neuron; GrRH: Gonadotropin Releasing Hormone; HPA: Hypothalamo-Pituitary Axis; HPG: Hypothalamo-Pituitary-Gonadal Axis; LH: Luteinizing Hormone; PKA: Phosphokinase A.

It is well known that stress can alter reproductive functions in both males and females (Kalantaridou et al., 2010). The biological mechanisms underlying the potential influence of the HPA axis on the functional output of the HPG axis (i.e., GnRH release) are illustrated in Figure 9, although other pathways have been proposed (Rivier and Rivest, 1991; Rivest and Rivier, 1995). Other factors that likely influence the HPG axis include the hypothalamic-pituitary-thyroid (HPT) axis, the animal’s nutrient status, photoperiod, and chronic stress levels. For example, it is known that high doses of atrazine reduce food intake and body weight, and these effects may confound or, in part, be responsible for the effects of the chlorotriazines on the HPA axis. These factors will not be discussed here.

**FIGURE 10:**
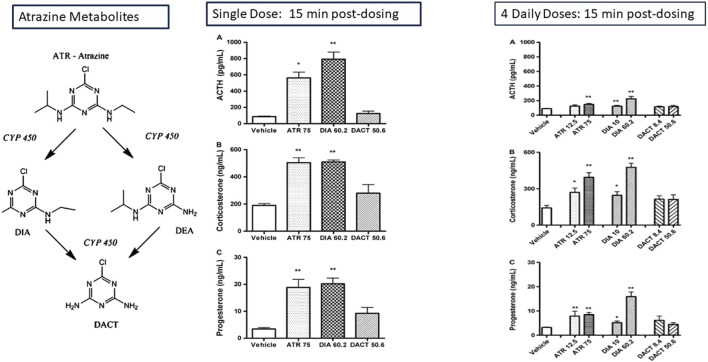
Effect of the Chlorotriazines on ACTH, corticosterone, and progesterone: Adaptation of the Adrenal Gland Response in Female LE Rats after Single or Multiple Equimolar Doses of the Chlorotriazines (Fraites et al., 2009). The effect of single and four daily equimolar equivalent doses of atrazine, DIA, DACT on plasma concentration of ACTH, corticosterone, and progesterone is shown 15 min post-dosing. A single dose of atrazine and DOA increases the plasma concentrations of the stress hormones ACTH, Corticosterone, and progesterone. DACT has no effect. After four daily doses of atrazine, the magnitude of the stress hormone response to atrazine is attenuated, although DIA’s response remains approximately the same. DACT remains ineffective in triggering a response. The effect of atrazine on progesterone levels is reduced by approximately one-half after 4 days of treatment, although the response of progesterone after 4 days is comparable to the effect observed after a single dose.

Atrazine (75 mg/kg/day) or DIA (60.2 mg/kg/day), when administered as a molar equivalent dose to atrazine by gavage to female LE rats, resulted in an increase in ACTH, corticosterone, and progesterone 15 min after treatment (Figure 10; Fraites et al., 2009). Five minutes of restraint in plastic cylinders also produced similar increases in pituitary and adrenal hormones. In contrast, a single dose or four daily doses of DACT did not affect adrenocorticotropic hormone (ACTH) or adrenal hormones, indicating that the most abundant chlorotriazine metabolite in the blood (Fraites et al., 2009; Laws et al., 2000; Simpkins et al., 2025) did not have the same effect on the HPA axis as parent atrazine or the monodealkylated metabolites, DIA or DEA. Laws et al. (2000) obtained similar results for a single, equimolar dose of atrazine (200 mg/kg), propazine, simazine, DIA, or DEA on ACTH, corticosterone, and progesterone in male Wistar rats. Again, DACT had no effect.

**FIGURE 11:**
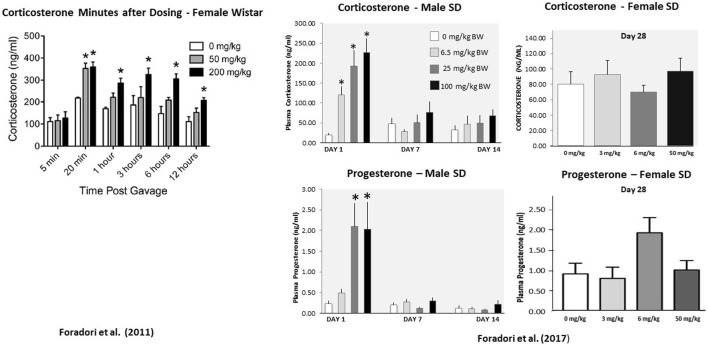
Time Course of the Effect of Atrazine on Adrenal Hormones in Female Wistar and Male and Female SD Rats. A single dose of 200 mg/kg atrazine results in a significant elevation in corticosterone and progesterone levels in female Wistar rats for up to 12 h post-dosing, while the effect of a dose of 50 mg/kg atrazine was attenuated 1 h post-dosing (left panel). After 28 days of treatment, there was no effect of atrazine on either corticosterone levels in female Sprague-Dawley rats at doses up to 50 mg/kg/day (Far right panel). Corticosterone levels were significantly increased in male SD rats after a single dose of atrazine as low as 6.5 mg/kg, but there was no effect on corticosterone in males after 7 or 14 daily doses (Middle panel). Progesterone was significantly elevated in males after single atrazine doses of 25 or 100 mg/kg, but there was no effect after a single dose of 6.5 mg/kg atrazine. Progesterone levels were comparable to those of control males after 7 or 14 days of atrazine treatment.

Foradori et al. (2011) and Foradori et al. (2017) demonstrated that adrenal hormones responded rapidly to high doses of atrazine, but the response disappeared after 7 days of treatment (Figure 11). This adaptation to the effect of atrazine may be due to changes in the central nervous system (CNS) or perhaps an upregulation of metabolism, leading to an increased rate of conversion of atrazine to the inactive form, DACT, after 4 days of treatment (Zimmerman et al., 2018).

**FIGURE 12:**
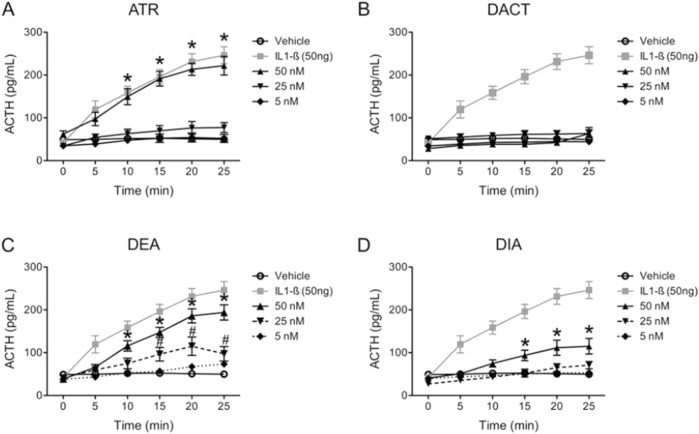
Effect of Atrazine, DEA. DIA or DACT injected into the Lateral Ventricle of the Brain on ACTH in Female SD Rats (Foradori et al., 2018). Experiment 6 from Foradori et al. (2018) determined the relative potency of atrazine, DEA, DIA, or DACT to induce an ACTH response in anesthetized female SD by directly injecting 5, 25, or 50 ƞM into the lateral ventricle of the brain. 50 ng of interleukin-1β (IL-1β) was employed as the positive control. The vehicle, which was artificial cerebrospinal fluid in which the chlorotriazine was solubilized, served as the negative control. The chlorotriazine concentrations employed were in the range of brain concentrations that were capable of activating the HPA axis following oral dosing (See Figure 10). The results indicate that atrazine was as potent as IL 1-B in inducing ACTH release **(A)**, DEA was second in potency, DIA was the least potent, and DACT had no effect. Blood collection procedures in this study were the same as described in Figure 8.

Foradori et al. (2018) reported that direct infusion of up to 50 nM of atrazine directly into the lateral ventricle of young adult female SD rats triggered ACTH release from the pituitary that was comparable in magnitude to the ACTH response to the administration of 50 ng of the positive control IL-1β (Figure 12A). DEA was almost as potent as atrazine (Figure 12C), and DIA was less potent than DEA (Figure 12D). DACT did not affect ACTH (Figure 12B).

Hotchkiss et al. (2009) examined the effect of atrazine on the anterior pituitary using a peri-fusion procedure in which the tissue was exposed directly to atrazine. A significant increase in ACTH release in response to corticotropin-releasing hormone (CRH, 10 μM) was observed, but there was no effect on ACTH release after 20-min exposure to 50 μM atrazine. Foradori et al. (2018) confirmed the results summarized by Hotchkiss et al. (2009) by showing that neither rat primary pituitary cells *ex vivo* nor mouse corticotrophs *in vitro* released ACTH when exposed for 30 min to concentrations of atrazine, DEA, DIA, or DACT ranging from 0.1 to 100 nM. The positive control substances (10 nM CRH or 5 nM 8-Br-cAMP) elicited an ACTH response in this model system. Foradori et al. (2018) showed that pre-treatment of female SD rats with dexamethasone, a synthetic glucocorticoid that suppresses ACTH release from the pituitary, blocked atrazine’s effect on corticosterone secretion from the adrenal gland. Surgically removing the pituitary did the same (Foradori et al., 2018).

Foradori et al. (2018) also demonstrated that the atrazine-induced ACTH response in female rats was inhibited by pretreatment with the CRH receptor antagonist astressin, thereby implicating CRH release in atrazine-induced HPA activation. However, unlike stress-induced HPA activation, a single dose of 100 mg/kg atrazine, while increasing plasma corticosterone, did not increase c-fos immunoreactivity in the periventricular nucleus. Collectively, these results indicate that atrazine’s effect on the HPA is likely at the level of the hypothalamus or elsewhere in the CNS but not in the adrenal gland.

**FIGURE 13:**
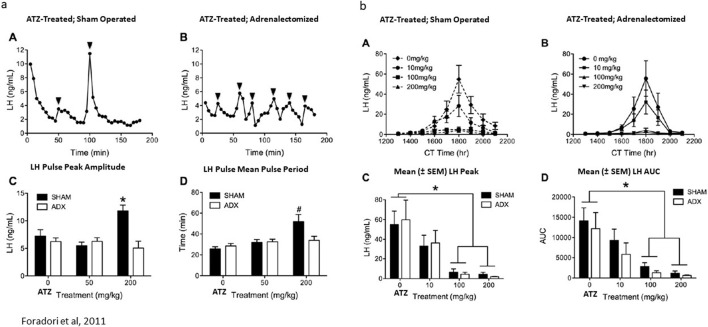
**(a)** Adrenalectomy Blocked Atrazine’s (200 mg/kg/day × 4 days) Effect on LH Pulse-Period and Pulse-Amplitude in Ovariectomized Wistar Rats (Foradori et al., 2011). The effect of 200 mg/kg atrazine on LH pulse period (A,C,D) was determined using the same techniques as described in Figure 8. The effect of adrenalectomy on the atrazine-induced effect on LH pulse period is shown in **(a)**, (B). Statistical analysis comparing sham-operated animals to adrenalectomized females is shown in (C,D). **(b)** Adrenalectomy had no effect on Atrazine’s Suppression of the LH surge in Ovariectomized Wistar Rats (Foradori et al., 2011). The dose-response effect of atrazine on the LH surge is shown in (B) after 4 days of treatment with atrazine at doses of 10, 100, or 200 mg/kg/day. Statistical analysis of peak LH amplitude (C) and area under the LH curve (AUC; (D)) in atrazine-treated groups indicates that atrazine doses of 100 and 200 mg/kg/day significantly reduced both parameters, yet the 10 mg/kg/day dose group was not significantly different from the control group. A subgroup of animals that had undergone adrenalectomy (B) displayed the same dose-response effect as the atrazine-treated groups that had their adrenal glands intact.

Finally, as described earlier, four daily doses of 200 mg/kg/day of atrazine resulted in increased LH pulse period and pulse amplitude in ovariectomized female Wistar rats (Figures 8B, 13a). Foradori et al. (2011) showed that adrenalectomy decreased LH pulse amplitude and pulse period in ovariectomized Wistar rats (Figure 13a). Adrenalectomy did not affect the dose-dependent reduction in the estrogen-primed LH surge in ovariectomized females (Figure 13b).

In summary, the rapid release of ACTH following administration of high doses of atrazine, DEA, and DIA, but not DACT to female SD rats, and the lack of effect of triazine exposure on the pituitary points to a hypothalamic or CNS site of action for the endocrine effect of the chlorotriazines on the HPA axis (Fraites et al., 2009). Cellular candidates for the site of action within the hypothalamus or midbrain are numerous, as are potential molecular targets. Additionally, interactions between the HPA and HPG axes are likely to occur. These interactions may ultimately be reflected in the altered GnRH pulse generator function in atrazine-treated, adrenalectomized female rats. The underlying mechanism, however, has not been established; interestingly, DACT does not alter the function of the HPA axis, yet it suppresses the LH surge (Minnema et al., 2001a). An important consideration for studies on the HPG and the HPA axis is whether the molecular target for atrazine is the result of an estrogen-like alteration of the cell function or some other molecular alteration that is not associated with an estrogen mode of action.

## 5 Interaction of atrazine with estrogen receptors

Steroid hormones, such as estrogen, mediate their physiological effects through classical estrogen receptors (ERs), including ERα and ERβ. Upon ligand binding and dimerization, these receptors translocate into the nucleus, where they directly regulate specific target genes by binding to estrogen-responsive elements (ERE). The structure and functions of ERs have been elucidated in detail (Nilsson and Gustafsson, 2000). Previous studies have also reported that estrogens are involved in carcinogenesis through the regulation of apoptosis, cell proliferation, and the cell cycle (Clusen et al., 2023; Toumba et al., 2024). Apart from classic ER-dependent genomic regulation, a non-genomic pathway has been discovered by which estrogen mediates cellular activities by binding to the G protein-coupled estrogen receptor (GPER) and directly triggers downstream cellular signaling events. The effect of the chlorotriazines on the classical estrogen receptors and the biosynthesis of estrogens is discussed next, and the effect of chlorotriazines on GPERs will be addressed in subsequent sections.

**FIGURE 14:**
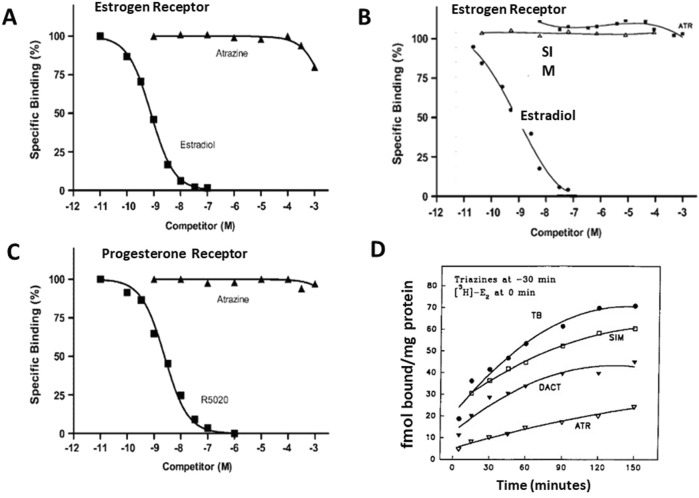
Estrogen and Progesterone Receptor Binding Assays for the Chlorotriazine **(A,B,D)**. Comparisons of competitive binding assays of atrazine and estradiol **(A)** from Cooper et al., 2007, and **(B)** Tennant et al., 1994b; **(C)** Competitive binding assay for atrazine and progesterone receptor (Cooper et al., 2007). **(D)** “Disequilibrium” assay after preincubation with atrazine, simazine, or DACT for 30 min prior to the addition of ^3^H estradiol. These interactions occurred only under conditions that most strongly favored the triazine (i.e., preincubation, a huge concentration excess, and chilling the tubes), which would question the integrity of the receptor protein. Atrazine appeared to compete more effectively than DACT or simazine.

Eldridge et al. (2008) evaluated the potential effects of atrazine and simazine on the estrogen receptors *in vitro* using ER competitive binding and ER transactivation assays of reporter genes (Supplementary Table S3). Although the majority of reports indicate that atrazine does not bind to the ER, even at very high concentrations (i.e., more than 100 mM) (Tennant et al., 1994b; Connor et al., 1996; 1998) (Figure 14). However, McMullin et al., 2004 reported that atrazine and DACT bound to ER with EC_50_s ranging between 10^−4^ and 10^−3^ M. Given the high concentration required to displace estradiol, however, these results may not accurately represent actual competitive binding, as the percent binding decreased from 100% at 10^−4^ M atrazine to zero at 10^−3^ M. The 100% decrease observed over one log unit of dose indicates that there may have been a problem with the binding kinetics (i.e., denaturing of the receptor). To date, there has not been a report indicating that atrazine, simazine, or related parent triazines or their metabolites either displaced estradiol in the competitive binding assay, nor have the triazines been shown to activate reporter genes responsive to ERα or ER*β* binding.

Several studies (Supplementary Table S4) have investigated the ability of atrazine to act as an estrogen agonist *in vivo* using the uterotrophic assay (OECD, 2007; USEPA, 2011). Tennant et al. (1994a) compared uterine weights in ovariectomized females dosed with atrazine or DACT (20, 100, or 300 mg/kg) or simazine (100 or 200 mg/kg) for three consecutive days with animals receiving the same dose of the atrazine or DACT and two ug estradiol (E2) (subcutaneous, sc) on the last 2 days. Uterine weights were increased in the positive controls receiving estradiol only, and the uterine weights of the females receiving 20 mg/kg atrazine or the two doses of simazine were not different from those of the estradiol-only treated females. In contrast, the uterine weights of the females co-treated with 100 and 300 mg/kg atrazine and DACT, as well as the 300 mg/kg dose of simazine, were significantly smaller than those of the estradiol-only controls, indicating a potential anti-estrogenic activity for atrazine. However, both the 100 and 300 doses of atrazine and DACT, as well as the 300 mg/kg dose of simazine, caused a significant reduction in body weight, raising a question about the specificity of this observation.

Tennant et al. (1994b) evaluated the amount of thymidine incorporation into uterine DNA in immature rats dosed with atrazine, simazine, or DACT (1, 10, 50, 100, or 300 mg/kg/day for 3 days) and dosed with 0.15 µg estradiol on day 2 of triazine treatment. The three higher doses resulted in a significant reduction in thymidine incorporation. Tennant et al. (1994b) also evaluated the concentration of progesterone receptors (PR) in the uteri of ovariectomized rats after treatment with estradiol alone or in combination with atrazine, simazine, or DACT (50 or 300 mg/kg/day for two consecutive days). There was no effect of atrazine on PR in the 50 mg/kg/day dose group compared to estrogen-treated females. The high-dose groups of atrazine and simazine (300 mg/kg/day) and, to a lesser extent, DACT displayed a significant reduction in the concentration of PR. These effects were accompanied by a significant loss in body weight in the high-dose groups compared to the vehicle control group.

Connor et al. (1996) challenged whether the effects reported by Tennant et al. (1994b) were mediated via the estrogen receptor. They evaluated the effect of chlorotriazines on several biomarkers for estrogenic activity in the uterus (wet weight, peroxidase activity and concentration). Immature rats were administered atrazine or simazine at doses of 50, 150, or 300 mg/kg for three consecutive days. Uterine wet weight was unaffected by treatment, although there was a significant reduction in cytosolic PR binding levels and uterine peroxidase activity. In contrast to the study by Tennant et al. (1994b), the reduction in PR induction and the estimate of peroxidase activity were highly variable and not dose-dependent. Differences between the protocols used in these two studies (ovariectomized vs. immature rats) and the doses of estrogen administered (2 vs. 10 μg/day) may account for the differences in results.

Yamasaki et al. (2000) examined the potential uterotrophic effects of atrazine on uterine weight, uterine fluid content, and its effect on the number of days to vaginal opening (VO), all of which are estrogen-sensitive biomarkers. Groups of rats were administered the positive control, 17*β*-estradiol (0 or 0.3 µg/day) or atrazine (0, 0.5, 5, or 50 mg/kg/day) for three or 7 days beginning on postnatal day 21. 17*β*-estradiol increased uterine weight and uterine fluid content but did not affect body weight or the number of days to VO. Atrazine at doses of up to 50 mg/kg/day had no uterotrophic effects, nor did it affect body weight or the number of days to VO.

Yamasaki et al. (2000) evaluated the anti-estrogenic potential of atrazine by comparing the effect of E2 alone on the biomarkers described above to the effects observed in rats that were co-administered E2 (0.3 μg/day) and atrazine (0, 0.5, 5, or 50 mg/kg/day). Atrazine, at a dose of 50/mg/kg/day, decreased the stimulatory effect of E2 on uterine weight and uterine fluid content, but there were no combined effects of atrazine + E2 on VO.

Eldridge et al. (1994) reported that high doses of atrazine (100 or 300 mg/kg/day) administered to female SD or F-344 rats for 14–23 days caused a significant dose-dependent reduction in the absolute and relative uterine weights in both strains of rats, again suggesting that high doses of atrazine may have anti-estrogenic properties.

Laws et al. (2000) evaluated the anti-estrogenic effect of atrazine in a pubertal development study in female Wistar rats. Atrazine administered by oral gavage daily from post-natal day (PND) 22 to PND 41, at doses of ≥ 50 mg/kg/day, caused a significant dose-dependent delay in the vaginal opening (VO). The no-observed-adverse-effect level (NOEL) was 12.5 mg/kg/day. Irregular estrous cycles were observed for 15 days after VO in the atrazine high-dose groups (100 and 200 mg/kg/day). The estrous cycles in these groups were characterized by prolonged periods of vaginal diestrus, indicating low endogenous estrogen levels. Similar results were observed in molar equivalent doses of DACT in a second study (Laws et al., 2003). It is known that normal development and pubertal timing of VO in rodents are dependent on estrogens as the hypothalamic-pituitary-ovarian (HPO) axis develops. Unfortunately, high doses of atrazine also reduce body weight gain postnatally, and this effect may contribute to delayed development, as observed in studies on pubertal development (Breckenridge et al., 2015). Laws et al. (2000) attempted to control for the significant reduction in body weight gain and reduced food intake in the 200 mg/kg/day atrazine-treated. They included a pair-fed control group that was provided the same amount of diet as the high-dose atrazine group. The number of days to VO in the pair-fed group was greater than in the control group, although the difference was not statistically significant.

Ashby et al. (2002) compared the effects of atrazine in Wistar and SD rats. Doses of 10, 30, and 100 mg/kg of atrazine were administered daily from PND 21 to PND 46. Uterine growth was delayed at PND 30 and 33 in Aderley Park (AP) rats exposed to 100 mg/kg atrazine, but the delayed development of the uterus was overcome by PND 43. VO was significantly delayed in AP rats for the 100 mg/kg ATR dose. By PND 46, VO was significantly delayed in SD rats exposed to both 30 and 100 mg/kg ATR, but uterine weights were unaffected by that time (as for AP rats). The NOEL in this study on sexually immature rats (10 mg/kg in SD; 30 mg/kg) was approximately the same as reported previously by Laws et al. (2000) in peripubertal Wistar rats (25 mg/kg).

In summary, atrazine did not transactivate the estrogen receptor in a wide range of *in vivo* and *in vitro* exposures, using various traditional estrogen-dependent endpoints during pubertal development. These findings essentially eliminate the hypothesis that atrazine induces mammary gland tumors by direct interaction with the classical ER_α_ or ER_*β*_ mode of action on the rat tissue. It is well known that estrogen can induce pseudoprecocious puberty, which has been associated with increased breast cancer risk (Goldberg et al., 2022). In these studies, very high doses of atrazine delayed pubertal onset, a result that is the opposite of what would be expected if the atrazine had estrogenic properties.

## 6 Interaction of atrazine with the g-protein coupled estrogen receptor (GPER)

GPER, previously known as GPR30, belongs to the G protein-coupled receptor superfamily. Carmeci et al. (1997) discovered this receptor in MCF-7 human breast cancer cells. Subsequently, it was demonstrated that GPER is not only located on the plasma membrane but also on the endoplasmic reticulum membrane and is ubiquitously expressed throughout the body (Revankar et al., 2005; Prossnitz and Arterburn, 2015).

**FIGURE 15:**
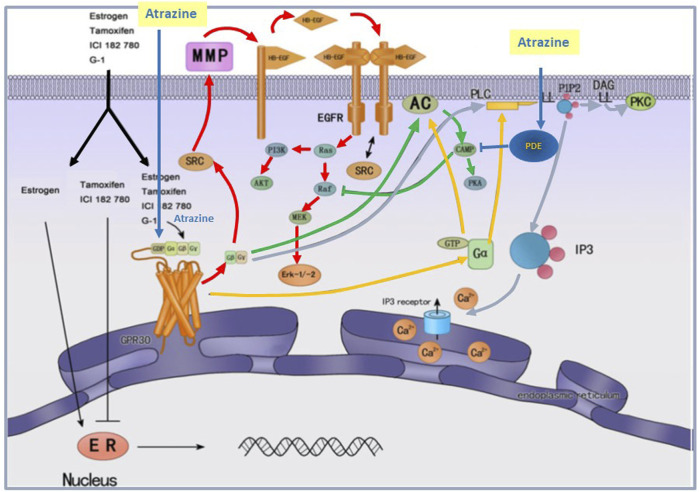
Schematic representation of the effect of Atrazine on Cyclic AMP and GPER-mediated signaling. Signal transduction pathways implicated in atrazine cellular bioactivity. At the cellular level, atrazine has been reported to affect multiple signal transduction pathways. These include activation of protein kinase A (PKA, green arrows), by inhibiting the inhibition of phosphodiesterase, and activation of the membrane-bound G protein estrogen receptor. Activation of cAMP may also enhance the activity of Erk1/2. If atrazine activates the GPER, it may also enhance Erk1/2 and PI3K, AKT activity through the EGFR receptor (red arrow) or by activating phospholipase, IP3 (calcium mobilization), or diacylglycerol (DAG) (blue arrows). It must be emphasized that the particular pathway affected may vary depending on the specific cell or tissue examined. Additionally, the responses elicited by a chemical may also be dose dependent, activating one pathway at a lower dose and recruiting other pathways as the dose is increased.

Filardo et al. (2000) demonstrated that estrogen-induced rapid activation of ERK1/2 was required for the expression of GPR30. Based on competitive binding assays, Revankar et al. (2005) and Thomas et al. (2005) demonstrated that E2 was the natural ligand for GPER. The activation of GPER, however, can also be viewed as potentially adverse in itself because such activation may trigger a range of downstream cellular events through intracellular kinases (Figure 15; ERK1/2, P13K and AKT (Madeo and Maggiolini, 2010; Qui et al., 2021)] that can lead to cell proliferation, enhanced survival of cancer cells, cell migration and metastasis (Figure 15; [Vivacqua et al., 2006a; b, Pandey et al., 2009; Prossnitz and Barton, 2011; Madeo and Maggiolini, 2010; Filardo, 2018; Qui et al., 2021]).

In addition to its role in promoting cancer, Ke et al. (2021) suggested that GPER can form a heterocomplex with kisspeptin one receptor (Kiss1R), resulting in reduced signaling by kisspeptin and decreased GnRH release. In rodents, this effect could potentially lead to a disruption of the estrous cycle, leading to an increased incidence of mammary tumors. Such an outcome might represent the molecular initiating event underlying the mammary tumor response observed in Sprague-Dawley rats (Simpkins et al., 2011). However, in this section, we will focus on the evidence that atrazine binds to GPER and activates signal pathways triggered by ligands for GPER (Figure 15). For example, GPER mediates the rapid non-genomic effects of estrogen, including the production of cyclic adenosine monophosphate (cAMP), the mobilization of intracellular calcium (Ca^2+^), transactivation of epidermal growth factor receptor (EGFR), and the activation of multiple kinases, such as phosphoinositide 3-kinase/protein kinase B (PI3K/AKT) and ERK1/2 mitogen-activated protein kinases (MAPK), often resulting in increased proliferation.

### 6.1 GPER ligand binding

The potential for atrazine to act as a ligand for the GPER was investigated by Thomas and Dong (2006), who transvected GPR30 into HEK293 cells. Competitive binding curves for atrazine vs. 17β estradiol were constructed from HEK293 plasma membranes. In this model, the IC_50_ for 17β-estradiol was 17.8 nM. The IC50 for atrazine could not be determined because atrazine did not displace 50% estradiol from GPR30 at concentrations up to 10 µM.

Albanito et al. (2015) performed ligand-binding studies using radio-labeled E2 as a tracer in ER-negative and GPER-positive SkBr3 breast cancer cells. Atrazine displaced the tritiated E2 ([H^3^] E2) from GPER in a concentration-dependent manner at concentrations ranging from 0.1 µM to 10 µM. In this model, at an E2 concentration of 0.1 μM, E2 was 100% bound to GPER, whereas 0.1 μM atrazine only displaced 20% of [H^3^] E2 from GPER. These results indicate that atrazine has a much lower affinity for GPER than does E2 in this model system. Simpkins et al. (2025) showed that, near the toxicological no-observed-adverse-effect level (NOAEL) for atrazine (∼3 mg/kg), the maximum plasma concentration of atrazine in rats was 36 ƞM. A comparable plasma concentration (40 ƞM) was obtained in female cynomolgus monkeys given a single oral dose of 2.5 mg/kg atrazine (Stuhler, 2014). These *in vivo* plasma concentrations at the NOAEL are more than 25 times lower than the lowest concentration tested by Albanito et al. (2015) *in vitro*. Humans exposed to atrazine in drinking water receive an oral dose of atrazine that is more than 25,000-fold lower than the toxicological NOAEL from *in vivo* studies in rodents (Simpkins et al., 2025). This suggests that the concentrations of atrazine tested in these *in vitro* model systems will not be attained in humans exposed to atrazine.

### 6.2 ERK1/2 phosphorylation

Albanito et al. (2015) reported that 1 μM atrazine activated ERK1/2 and the expression of estrogen target genes, which are associated with both GPER and ERα receptor expression. These effects were blocked by silencing the expression of GPER or ERα.

### 6.3 mRNA expression in estrogen-targeted genes

Albanito et al. (2015) reported, based on densitometric analyses, that exposure of BG-1 cells to atrazine for 1 h at a concentration of 1 μM/L significantly increased the expression of mRNA for c-fos, CTGF, and cyclin A compared to the DMSO vehicle. After 24 h of exposure to atrazine, mRNA for the progesterone receptor (PR), pS2, and Cyclin D1 were also increased. Exposure of BG-1 cells to 100 ƞM/L E2 for 1 or 24 h increases mRNA expression for all these genes, as well as for Cathepsin D and Cyclin E.

#### 6.3.1 Upregulation of c-fos gene expression at the protein level

Albanito et al. (2015) reported, based on Western blots and densitometric analysis, that exposure to 1 uM/L atrazine or 100 nM/L E2 increased the amount of c-fos protein in BG-1 and 2008 human ovarian cancer cells. These effects were nullified by the presence of an ER Antagonist (ICI), an EGFR Inhibitor (AG), a MEK kinase inhibitor (PD), a protein kinase C inhibitor (GFX), and a phosphoinositide 3-kinase inhibitor (WM).

#### 6.3.2 Dose-dependent effect of BG-1 and 2008 cell proliferation

Albanito et al. (2015) reported that exposure of BG-1 and 2008 human ovarian cancer cells to atrazine or E2 at concentrations ranging from 1 nM/L to 1 uM/L caused a dose-dependent increase in cellular proliferation. Co-exposure to the EGFR Inhibitor (AG) or the MEK kinase inhibitor (PD) nullified the proliferative response. Prior transfection of the cells with SiRNA (small interfering RNA) for E2 or shGPER (short hairpin RNA for GPER) also blocked the proliferative response. In contrast, Chevalier et al. (2016) reported that exposing JKT-1 cells derived from human testicular seminomas to atrazine for 24 h resulted in a significant reduction in cell proliferation at concentrations ranging from 10^−5^ M to 10^−10^ M.

#### 6.3.3 Cellular migration of cancer-associated fibroblasts (CAFs)

Albanito et al. (2015) reported that exposure of CAFs to 1 μM of atrazine or 1 nM of E2 caused an increase in cell migration. Prior transfection of CAFs with shGPER or shCTFG (short hairpin RNA for CTFG) blocked cellular migration response to atrazine or E2 exposure *in vitro*. Similar effects have been reported in mouse 4T1 breast cancer cells (Wang et al., 2023); H22 liver cancer cell lines (Tian et al., 2018); Skov3 and A2780 human epithelial cancer cells (Chen et al., 2021); and RM1 prostate cancer cell line (Hu et al., 2016) at atrazine concentrations ranging from 0.01 to 1.0 uM. It is unclear whether the reported effects of atrazine are mediated through the GPER and whether any adverse effects accrue *in vivo* as a result of atrazine or its chloro-metabolites binding to GPER.

## 7 Effects of atrazine on steroidogenesis via aromatase

Aromatase (CYP19) is the rate-limiting enzyme in the conversion of androgens to estrogens. Cyclic nucleotide PDE controls the rate of breakdown of the intracellular messengers, 3′, 5′-cyclic adenosine (cAMP), and guanosine monophosphates (cGMP) to their respective (AMP or GMP) bi-products. Steroidogenic CYPs are induced by synthetic cAMP analogs or stimulation of adenylate cyclase with forskolin, resulting in increased intracellular cAMP levels. Inhibition of PDE increases cyclic AMP levels, which, in turn, stimulate the cAMP-mediated protein kinase A pathway, leading to increased gene transcription (Rainey et al., 1993; Staels et al., 1993).

Several *in vitro* studies reviewed by Simpkins et al. (2025) have investigated the effect of atrazine, DEA, DIA, and DACT on PDE (Roberge et al., 2004; Simpkins et al., 2025), cyclic AMP (Kucka et al., 2012; Sanderson et al., 2001b); aromatase mRNA expression (Sanderson et al., 1999; 2002; Simpkins et al. (2025) and aromatase catalytic activity (Sanderson et al., 2001a; Higley et al., 2010). These studies used steroidogenic cell lines, such as the H295R human adrenocortical carcinoma cell (Staels et al., 1993; Rainey et al., 1993), which are incapable of metabolizing atrazine (Simpkins et al., 2025). In the following section, we will review the concentration-response relationships for the effects of atrazine, DEA, DIA, and DACT on PDE, cyclic AMP, and aromatase to determine if altered steroidogenesis is likely to occur *in vivo.*

### 7.1 Effects of the chlorotriazines on phosphodiesterase *in vitro*

Roberge et al. (2004) evaluated whether atrazine DEA, DIA, or DACT inhibited PDE, the enzyme that converts cAMP to 5′-AMP. Atrazine had an IC_50_ of 1.8 μM for PDE inhibition compared to IBMX, which had an IC_50_ value of 4.6 μM. Atrazine’s chloro-metabolites, DEA, and DIA were less potent than atrazine, and DACT was the least effective. IC_50_ values for atrazine’s chloro-metabolites were not calculated because of their low aqueous solubility at concentrations above 230 µM.

Simpkins et al. (2025), who solubilized atrazine and its metabolites in DMSO, found that atrazine’s IC_50_ for PDE inhibition was 39.5 μM compared to IBMX’s IC_50_ of 1.7 μM. The IC_50_ values for DIA (1,371 μM) and DEA (7,156 μM) were 35 and 181 times less potent inhibitors of PDE than atrazine. DACT (IC50 = 27.4 M), hydroxyatrazine, and ammeline were virtually inactive. Simpkins et al. (2025) reported that when DACT was added to the media with atrazine at concentrations as low as 0.5 ƞM, the IC_50_ for atrazine was increased approximately 10-fold, indicating that atrazine was a less potent inhibitor of PDE in the presence of DACT. They concluded that DACT, while inactive as a PDE inhibitor, blocked atrazine from reaching a critical site on PDE that was involved in converting cAMP to 5′-AMP. Simpkins et al. (2025) showed that PDE4 inhibition observed *ex vivo* was likely not relevant *in vivo.* They found that the area under the curve (AUC) of plasma concentrations of the chlorotriazines in female SD rats following gavage or dietary administration at the point of departure dose of 2.56 mg/kg/day was 237 to 686-fold lower than the concentration needed to inhibit PDE4 in H295R cells, respectively. This reduction in potency was attributed to the inability of H295R cells to metabolize atrazine to DACT, the predominant chlorotriazine metabolite *in vivo* (∼95%) at steady state. Interestingly, when Roberge et al. (2006) evaluated the effect of atrazine on hepatocytes from swine, which are capable of metabolizing atrazine, he found that exposure to atrazine did not affect PDE.

### 7.2 Effects of the chlorotriazines on aromatase mRNA expression *in vitro*

Sanderson et al. (1999) reported that 30 µM of atrazine, simazine, or propazine increased the expression of aromatase mRNA in H295R cells by approximately 150, 175, or 200% compared to the CYP 19 beta-actin controls. Simpkins et al. (2025) found that H295R cells exposed to 10 µM of atrazine for 2–72 h increased aromatase mRNA expression more than 2-fold. A transient, significant increase was observed in cells exposed to 1 µM atrazine for 24 h, but not at any other time point. In contrast, 0.1 µM atrazine had no effect at any time point through 72 h.

### 7.3 Effects of the chlorotriazines on cyclic AMP *in vitro*

The association between atrazine exposure and cAMP levels had been noted earlier by Messner et al. (1979) and Leroux et al. (1999). Messner et al. (1979) described the effects of *in vivo* (diet or i.p. injections) atrazine on liver glycogen metabolism. They observed increased cyclic AMP in the liver, increased liver glycogen phosphorylase activity, decreased liver glycogen content, and increased blood glucose concentrations. In a follow-up *in vitro* study using hepatocytes, they reported that triazines did not affect adenylate cyclase activity. However, PDE was inhibited non-competitively. When atrazine was added to isolated hepatocytes, it was noted that the stimulatory effect of glucagon on cAMP accumulation was potentiated. Thus, it was plausible that atrazine inhibited PDE *in vitro*. The authors also reported that simazine, propazine, or prometryn elevated the level of cyclic AMP in the liver. The doses used in this study were extremely high (250 mg/kg). Leroux et al. (1999) tested synthetic triazine derivatives for their tracheal smooth muscle relaxant property and observed that they increased cAMP concentration with varying potencies.

Sanderson et al. (2002) noted that atrazine (30 μM) increased cAMP levels in H295R cells, and the response time and peak concentration of cAMP, as well as the return to baseline levels, were similar to those observed after treatment with the non-selective PDE inhibitor, isobutyl methylxanthine (IBMX; 100 μM). These investigators went on to suggest that the time-response profiles for aromatase and CYP19 mRNA induction by atrazine and 8Br-cAMP were comparable and that triazines may induce changes in aromatase by increasing transcription of the CYP19 gene and increasing aromatase availability.

### 7.4 Effects of the chlorotriazines on aromatase activity *in vitro*

Sanderson et al. (2001a) exposed H295R cells *in vitro* to atrazine, simazine, and propazine at concentrations ranging from 0 to 30 µM. The three triazines induced an increase in aromatase activity (picomoles of androstenedione converted per hour per milligram cellular protein) in H295R cells after 24 h. The induction was concentration and time-dependent, with an apparent maximum of about 2.5-fold at 30 μM. Sanderson et al. (2001a) demonstrated that atrazine and its mono-dealkylated metabolites (DEA and DIA) also increased aromatase activity. The di-dealkylated metabolite (DACT) was inactive at concentrations up to 30 µM. Sanderson et al. (2001a) compared aromatase activity in H295R cells, JEG-3 placental cells, and MCF-7 breast cancer cell lines after exposure to chlorotriazine *in vitro*. These cell lines were chosen to compare the effect of atrazine on aromatase activity in cells that express aromatase at relatively high levels (JEG3 cells) to cells where the enzyme expression is very low (MCF-7 cells). Atrazine, simazine, and propazine induced a concentration-dependent increase in aromatase activity above 1 μM. As in H295R cells, DIA and DEA displayed an increase in aromatase activity in the JEG-3 cell line at a concentration of 30 μM, but DACT was inactive. Aromatase activity and mRNA aromatase expression were undetectable in MCF-7, and they did not respond to exposure to any of the chlorotriazines.

### 7.5 Role of steroidogenic factor-1 in aromatase response to atrazine

In the preceding section, we reviewed the evidence supporting the hypothesis that atrazine affects steroidogenesis by inhibiting PDE, resulting in the accumulation of cyclic AMP, induction of aromatase mRNA, and increased aromatase activity. An alternative hypothesis proposed by Fan et al. (2007a) and Fan et al. (2007b) is that atrazine binds to steroidogenic factor 1 (SF-1), a nuclear receptor found in steroidogenic cells (e.g., adrenocortical, granulosa, and Leydig cells), which, when activated, increases aromatase gene expression and aromatase activity. SF-1 is a ligand for the NR5A nuclear orphan receptor that regulates several components of the endocrine and reproductive systems (Meinsohn et al., 2019). SF-1 (also known as NR5A1) is mainly expressed in steroidogenic tissues. In contrast, NR5A2 is expressed in tissues of endodermal origin, as well as in the gonads. Both receptors regulate the homeostasis of cholesterol and steroidogenesis, as well as cell proliferation and stem cell pluripotency, through interactions with cofactors. NR5A2, also known as liver receptor homolog-1 (LRH-1), can recognize the same DNA binding sites, but it elicits divergent effects depending on the target tissues and cells.

Fan et al. (2007a) transfected H295R cells with an aromatase luciferase reporter gene (pGL3-ARPII4), either excluding or including the transfection of human SF-1. Exposure of H295R cells with the transfected luciferase reporter gene to 10 µM atrazine or simazine increased relative luciferase activity approximately 2.5-fold for atrazine and 2.3-fold for simazine. This response was comparable to the results obtained by Sanderson et al. (2001a), who reported that exposing H295R cells to 10 µM atrazine or simazine for 24 h increased aromatase activity more than 2.2-fold, using the more traditional tritiated water method of Lephart and Simpson (1991) to quantify aromatase activity.

Morinaga et al. (2004) reported that ovarian granulosa cells (KGN cells) exposed to 10 µM atrazine or simazine did not exhibit any increase in aromatase activity, as measured using the tritiated water method. Fan et al. (2007b) postulated that this was because the number of SF-1 mRNA copies in KGN cells was 54 times lower than that observed in atrazine-responsive H295R cells. Fan et al. (2007b) transfected KGN cells with SF-1 and exposed them to 10 µM atrazine or simazine for 24 h. SF-1-transfected KGN cells now displayed increased aromatase activity, which was comparable to H295R cells. Furthermore, both atrazine and simazine induced an aromatase response when SF-1 and the promoter were co-transfected into NIH3T3 mouse embryonic fibroblast cells. However, when only the PII promoter was transfected into NIH3T3 cells, no response was observed. Fan et al. (2007b) suggested that although atrazine may increase aromatase levels in H295R cells *in vitro* by inhibiting PDE, they postulated that atrazine also binds to SF-1 and induces aromatase expression via promoter II.

Suzawa and Ingraham (2008) also studied the effect of atrazine on aromatase activity. Their study examined both zebrafish and mammalian cell lines to identify the affected genes and potential transduction pathways following atrazine exposure to zebrafish *in vivo* and various cell lines *in vitro*. Using JEG3 human placental cells, which contain modest amounts of SF-1, they found that atrazine treatment (10^−7^ to 10^−5^ M) activated the aromatase promoter in a dose-dependent manner. In contrast, no activation was observed when the parent reporter was examined with a mutant SF-1, suggesting that atrazine’s effect depends on DNA binding and receptor occupancy of the promoter, as suggested previously by Fan et al. (2007a) and Fan et al. (2007b). In a second series of experiments aimed at discerning the transduction pathways associated with atrazine activation of aromatase, Suzawa and Ingraham (2008) demonstrated that inhibitors of both mitogen-activated protein kinases (MAPK) and phosphatidylinositol 3-kinases (PI3K) decreased or eliminated atrazine-induced aromatase activation.

These results are consistent with atrazine’s rapid activation of the MAPK pathway, which may be in response to PDE inhibition or to binding to the G-protein-coupled estrogen receptor (GPER). Consistent with this response, Suzawa and Ingraham (2008) demonstrated that the activation of MAPK was followed by peak phosphorylation of SF-1 and activation of the PI3K pathway as determined by phosphorylation of Akt/PKB. JEG3-responsive cells exposed to atrazine at a concentration of 1 μM, as observed by Suzawa and Ingraham (2008), showed a significant and consistent increase in cellular cAMP; however, the response was significantly less pronounced than that observed with forskolin. Collectively, these results suggest that although atrazine may not directly interact with NR5A receptors, it may activate three signaling pathways that are known to activate NR5A receptors, including the phosphorylation of SF-1, the generation of SF-1 ligands, and the increased production of cAMP.

## 8 Effects of the chlorotriazines on ovarian granulosa cells function *in vitro* and *in vivo*

The development of ovulatory follicles involves the differentiation of their granulosa cell component from a mitotically active, estrogen-secreting type (immature cells) into a nondividing, luteinized, progesterone-secreting type (mature cells). Granulosa cell differentiation is regulated by two pituitary hormones, FSH and LH, as well as intra-ovarian steroids and growth factors. FSH activates three major signaling pathways in immature granulosa cells, namely, cAMP/protein kinase A, ERK1/2, and PKB (Wayne et al., 2007; Hunzicker et al., 2012; Fan et al., 2007a; b). In the immature granulosa cell, the primary signaling cascade regulated by FSH involves the activation of adenylyl cyclase, leading to the production of cAMP, which, in turn, activates PKA, resulting in the phosphorylation of key substrates, including the cAMP response element-binding protein (CREB) (Gonzalez-Robayna et al., 1999). The cAMP cascade is involved in the transcription of the luteinizing hormone receptor (LHR) (Chen et al., 2000) and CYP19A, leading to enhanced estradiol production (Fitzpatrick et al., 1997), which in turn affects markers of proliferation and differentiation of granulosa cells. However, others have shown that the FSH-driven responses are more complex, involving the activation of the PKB and ERK1/2 signaling pathways (Wayne et al., 2007; Zeleznik and Saxena, 2003; Pogrmic-Majkic, 2018). In pre-ovulatory granulosa cells, the primary signaling cascade is initiated by the activation of ERK1/2 by the phosphorylation of human chorionic gonadotropin (hCG). Pogrmic-Majkic and her colleagues (Pogrmic-Majkic et al., 2014; Pogrmic-Majkic et al., 2016; Fa et al., 2013) conducted a series of studies to evaluate the effect of atrazine on endocrine and signal transduction pathways, aiming to identify its impact on these processes.

Fa et al. (2013) evaluated the effect of atrazine on ovarian granulosa cell cultures prepared from immature (21–24 days old) Wistar rats. Three markers of FSH- or 8-Br-cAMP-stimulated immature granulosa cells were assessed: Cyp19a1 mRNA, LHR mRNA, or estradiol secretion into the media. Atrazine alone, at doses of 20 or 40 μM, was without effect on any of the three endpoints. However, when the cells were incubated with FSH + atrazine, both the 20 and 40 μM doses of atrazine blocked the increase in estradiol. This was not the case for 8-Br-cAMP, in which both doses of atrazine increased the concentrations of estradiol in the median over that observed with FSH alone. These results revealed a distinction between the effect of stimulating the membrane receptor versus activating the post-receptor cAMP response. The same distinction between FSH receptor stimulation and cAMP activation was also observed for the expected increases in Cyp19a1 mRNA and LHR mRNA following combined treatment with atrazine and FSH or atrazine and 8-Br-cAMP. Atrazine blocked the FSH stimulation of these two endpoints but did not block the increase observed after 8-Br-cAMP, again demonstrating a difference between FSH receptor activation and bypassing the receptor with 8-Br-cAMP. An obvious limitation of these studies is the high doses of atrazine used (20 and 40 μM) and the lack of demonstration that granulosa cells can metabolize atrazine. As such, these studies are unlikely to be relevant to human exposure to atrazine. Such studies, however, illustrate the difficulties of extrapolating findings from transformed cell lines to the more complex, but defined pathways present *in vivo*.

Fa et al. (2013) demonstrated that the atrazine-induced reduction in estradiol, CYP19a1, and LHR was blocked when the cells were pretreated with U0126, a pharmacological inhibitor of the ERK1/2 signal pathway, indicating a role for this pathway as a prerequisite for the atrazine effect. The inhibitory effect of atrazine on markers of granulosa cell maturation (including increased aromatase) is in contrast to the stimulatory effect noted in JEG3 cells by Suzawa and Ingraham (2008). Fan et al. (2004) also demonstrated the significance of this pathway for the LH-induced oocyte resumption of meiosis, ovulation, and luteinization of the follicle. Fa et al. (2013) proposed that FSH activates both the cAMP and ERK1/2 pathways and that atrazine targets a yet-to-be-identified transcriptional factor that prevents the cAMP-driven expression of LHR mRNA.

Pogrmic-Majkic et al. (2014) examined the effect of atrazine on the transition from the immature to preovulatory follicular stage in immature rat granulosa cells. Immature granulosa cells were treated with FSH to trigger *in vitro* proliferation and differentiation of granulosa cells, and the effect of atrazine on endocrine and signal transduction pathways critical to granulosa cell development was evaluated. They observed that high concentrations of atrazine (20 µM) caused dysfunction in the cAMP, AKT, and Erk1/2 pathways. Their data support the hypothesis that the effects of atrazine are initiated by the inhibition of PDE4, which is the primary regulator of cAMP, AKT, and ERK1/2 signaling pathways. Changes in the properties of these signaling pathways represent the initial step in a complex modification of granulosa cell function, leading to premature luteinization.

Pogrmic-Majkic et al. (2018) investigated the effect of atrazine on granulosa cell function in human primary cell cultures. Human cumulus granulosa cells were obtained from women undergoing *in vitro* fertilization. Human granulosa cells were exposed to 20 μM atrazine, a dose previously shown to alter steroidogenesis in granulosa cells from immature rats. Atrazine inhibited the steroidogenic process in human cumulus granulosa cells by suppressing FSH-induced CYP19A1 mRNA expression and progesterone production. Atrazine also diminished the FSH-induced expression of the ovulatory genes amphiregulin and epiregulin. Thus, as in the rat granulosa cells, atrazine had an anti-steroidogenic action *in vitro* following FSH stimulation (Fa et al., 2013) or after *in vivo* exposure of immature rats to gonadotropins and atrazine (Samardzija et al., 2016).

Pogrmic-Majkic et al. (2018) also showed that atrazine increased the activity of PDE and decreased cAMP levels in the FSH and forskolin-simulated human cumulus granulosa cells. These results are in direct contrast to those of Roberge et al. (2004), who demonstrated that atrazine inhibited PDE *ex vivo.* The results are also in contrast to those found by Sanderson et al. (2001b) who reported that 30 µM atrazine increased cAMP levels in H295R cells, and Kucka et al. (2012), who found an approximate 2 to 3-fold increase in cAMP levels in pituitary cells exposed to atrazine *in vitro* at concentrations greater than 10 µM. Pogrmic-Majkic et al. (2018) noted that PDE3, PDE4, PDE7, and PDE8 expression was greatest in the human ovary. PDE3 was primarily found in the oocyte, while PDE4 and PDE8 were significantly expressed in granulosa cells of late-stage follicles and after ovulation. They speculated that variations in the PDE isoform-specific expression and activity might account for the observed differences.

## 9 Epidemiological studies on breast, ovarian, and uterine cancers

Published epidemiological studies on the association between atrazine exposure and the incidence of breast (Supplementary Table S5a) and ovarian (Supplementary Table S6a) cancers were reviewed by Sathiakumar et al. (2011) and Boffetta et al. (2013). There were no published data on uterine cancer. In the following analyses, we relied on the reviews by Sathiakumar and Boffetta to identify studies published before 2013. We search PubMed for new epidemiological studies on atrazine or the chlorotriazines. We found three publications that reported data on the association between atrazine use and the incidence of breast (Remigio et al., 2024) and ovarian cancer (Inoue-Choi et al., 2016; Renier et al., 2024). To facilitate the visualization of the data, we summarized risk ratios (RR) and their 95% confidence intervals for all cancer incidence studies. Overall, as previously concluded by Sathiakumar et al. (2011) and Boffetta et al. (2013), there is no compelling evidence of an association between atrazine exposure and the incidence of breast cancer (Supplementary Table S5b) or ovarian cancer (Supplementary Table S6b) in women.

## 10 Discussion

There is clear and consistent evidence that atrazine alters the development of mammary gland tumors in the female SD rats by causing premature reproductive aging in SD but not Fischer-344 rats. Several independent laboratories have confirmed that the effect of atrazine on mammary tumors in SD rats is related to the suppression of the ovulatory LH surge, resulting in a prolongation of the number of days the animal spends in estrus. The molecular initiating event (MIE) underlying the suppression of the LH surge remains unclear. However, the MIE likely involves altering the function of the neuronal circuitry in the hypothalamus that regulates pulsatile GnRH or the GnRH surge mechanism in rodents, perhaps through direct or indirect effects on the KnDY neurons in the arcuate nucleus, kisspeptin neurons RP3V, or the GnRH neuron itself. The interaction between HPG and the HPA axes, while interesting, does not appear to be central to the effect of atrazine on the GnRH because the HPA axis effects are short-lived, and DACT, which suppresses the LH surge, does not affect the HPA axis.

In considering alternate modes of action, there is strong *in vitro* and *in vivo* evidence indicating that atrazine does not alter the transactivation of the genomic estrogen receptors (ERα or ER*β*) and thus does not promote cancer by this mode of action.

Exposure of specific cancer cell lines to atrazine *in vitro* has been reported to bind to and transactivate GPER, which is located on the cell plasma membrane or intracellularly on the endoplasmic reticulum. However, in other transformed cell lines and studies on primary cell cultures, opposite effects have been reported. These *in vitro* studies, however, all suffer from the fact that exposure is typically in concentration ranges that are not encountered *in vivo,* due to the rapid metabolism and clearance of atrazine (See Simpkins et al., 2025).

*Some in vitro* studies have reported that atrazine increases cell proliferation, impairs apoptotic mechanisms, or alters the cell cycle by binding to GPER. However, other studies using the same approach in different cell lines, or primary cell cultures obtained from rats or humans, demonstrate that atrazine decreases steroidogenesis, proliferation, and enhances apoptosis. Competitive binding assays evaluating the affinity of atrazine for the GPER demonstrated either no binding or binding in the µM range, concentrations that are much higher than needed for estradiol transactivation. To our knowledge, the potential for atrazine to bind to the GPER in mediating any effect of atrazine *in vivo* has not been reported.

Atrazine has been shown to increase aromatase activity *in vitro*, thereby increasing the conversion of androgens to estrogen. The mechanism underlying this effect is related to the inhibition of PDE, which converts cyclic AMP to AMP. The buildup of intracellular cyclic AMP levels could result in increased aromatase activity, triggering a multitude of other cyclic AMP–dependent signaling pathways. However, when metabolically competent cells, such as hepatocytes, are exposed to atrazine *in vitro*, there are no effects of atrazine on PDE (Roberge et al., 2006). This finding is consistent with the absence of effects of atrazine *in vivo* on androgen- (Hershberger Assay) or estrogen- (Uterotrophic assay) dependent tissues. It is also consistent with the absence of mammary tumors in ovariectomized, atrazine-treated SD rats.

Collectively, the interpretation of alternative MOAs, the lack of relevance of the *in vivo* carcinogenicity studies to humans, and the null epidemiological studies indicate that atrazine and its metabolites are unlikely to cause endocrine-related cancers in human females.

## Glossary

ACTH: adrenocorticotropic hormone
AKT: protein kinase B
AUC: area under the curve
AVP: arginine vasopressin
PKB: protein kinase B
AVPN: anteroventral periventricular nucleus
CRH: corticotropin-releasing hormone
CNS: central nervous system
cAMP: cyclic adenosine monophosphate
CREB: cAMP response element-binding protein
DACT: diamino-chlorotriazine
DEA: deethylatrazine
DIA: deisopropylatrazine
EGFR: epidermal growth factor receptor
E2: 17β-estradiol
ER: estrogen receptor
ERE: estrogen-responsive elements
F-344: Fischer-344
FSH: follicle-stimulating hormone
FMP: final menstrual period
GnRH: gonadotropin-releasing hormone
GPER: G-protein-coupled estrogen receptor
GPR30: G-protein coupled estrogen receptor 30
cGMP: cyclic guanosine monophosphate
HPA: hypothalamic-pituitary-adrenal
HPG: hypothalamic-pituitary-gonadal
HPO: hypothalamic-pituitary-ovarian
HPT: hypothalamic-pituitary thyroid
KNDy: kisspeptin, neurokinin B, and dynorphin-containing
Kiss1R: kisspeptin one receptor
LE: Long-Evans
LH: luteinizing hormone
LHR: luteinizing hormone receptor
MAPK: mitogen-activated protein kinases
MOA: modes of action
NOEL: no-observed-adverse-effect level
NOAEL: no-observed-adverse-effect level
PDE: phosphodiesterase
PI3K: phosphatidylinositol 3-kinases
PKA: protein kinase A
PLC: phospholipase C
PMD: Post-natal day
POA: preoptic nucleus
PR: progesterone receptor
RP3V: rostral periventricular areas of the third ventricle
SD: Sprague Dawley
SAR: structure-activity relationship
sc: subcutaneous
SCN: suprachiasmatic nucleus
SiRNA: small interfering RNA
shGPER: short hairpin RNA for GPER
SF-1: steroidogenic factor 1
TRH: thyrotropin-releasing hormone
VO: vaginal opening
VIP: vasoactive intestinal peptide
