# Supplementary figures and images for: Effects of atrazine on the HPG and HPA axes and steroidogenic pathways in females: relevance to reproductive function and breast, ovarian and uterine cancer
Source: Front Toxicol. 2026 Jan 5;7:1686703. doi: 10.3389/ftox.2025.1686703 (PMC12812897; doi:10.3389/ftox.2025.1686703)

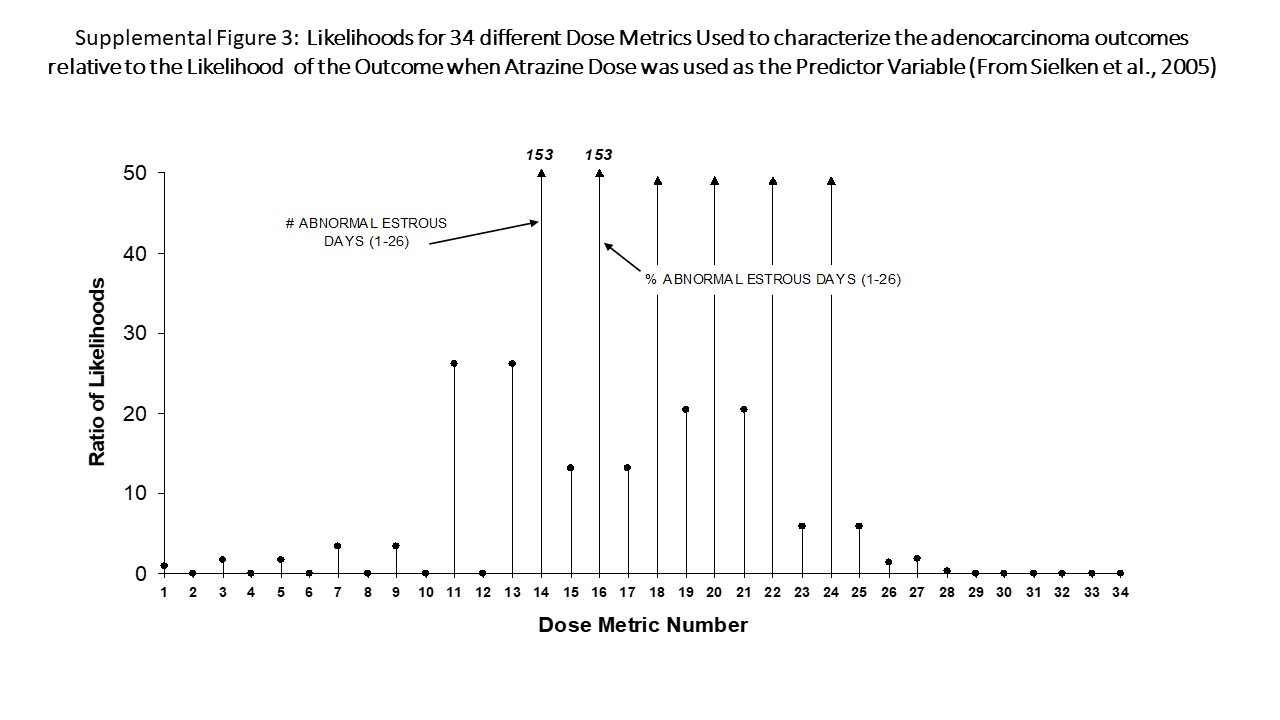

Supplement: Supplementary file 1 [file Image3.jpeg]

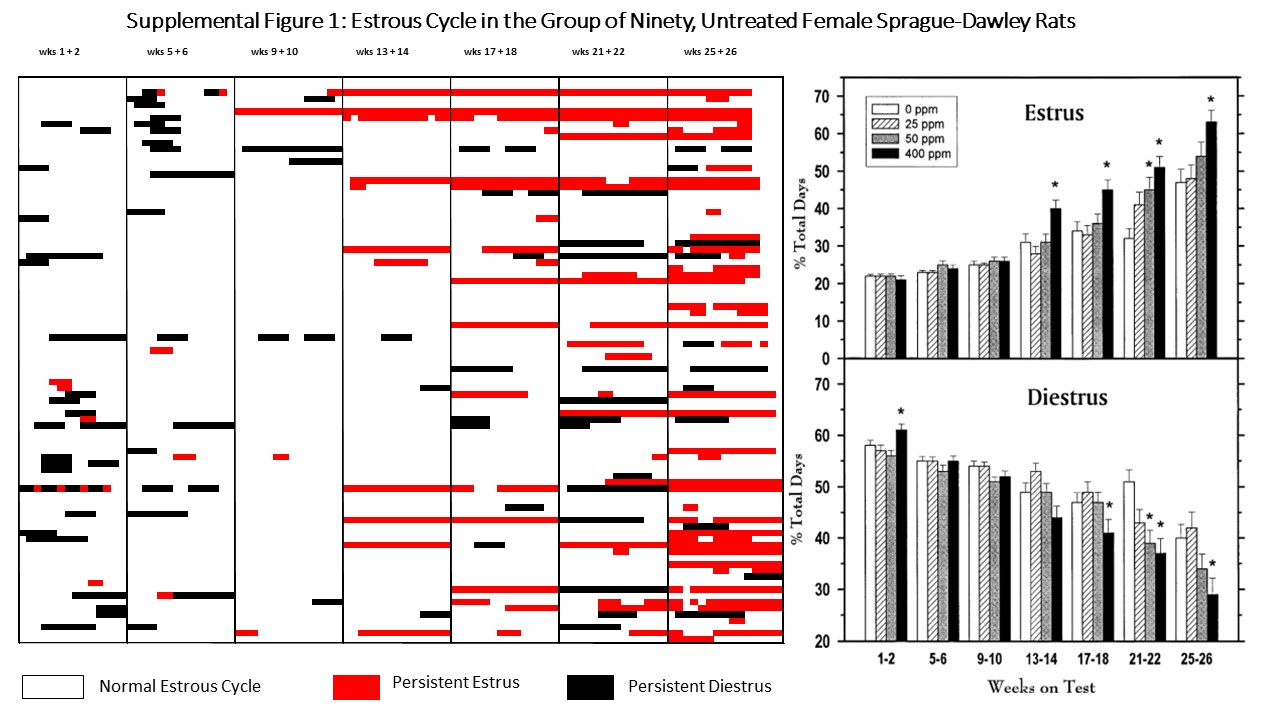

Supplement: Supplementary file 3 [file Image1.jpeg]

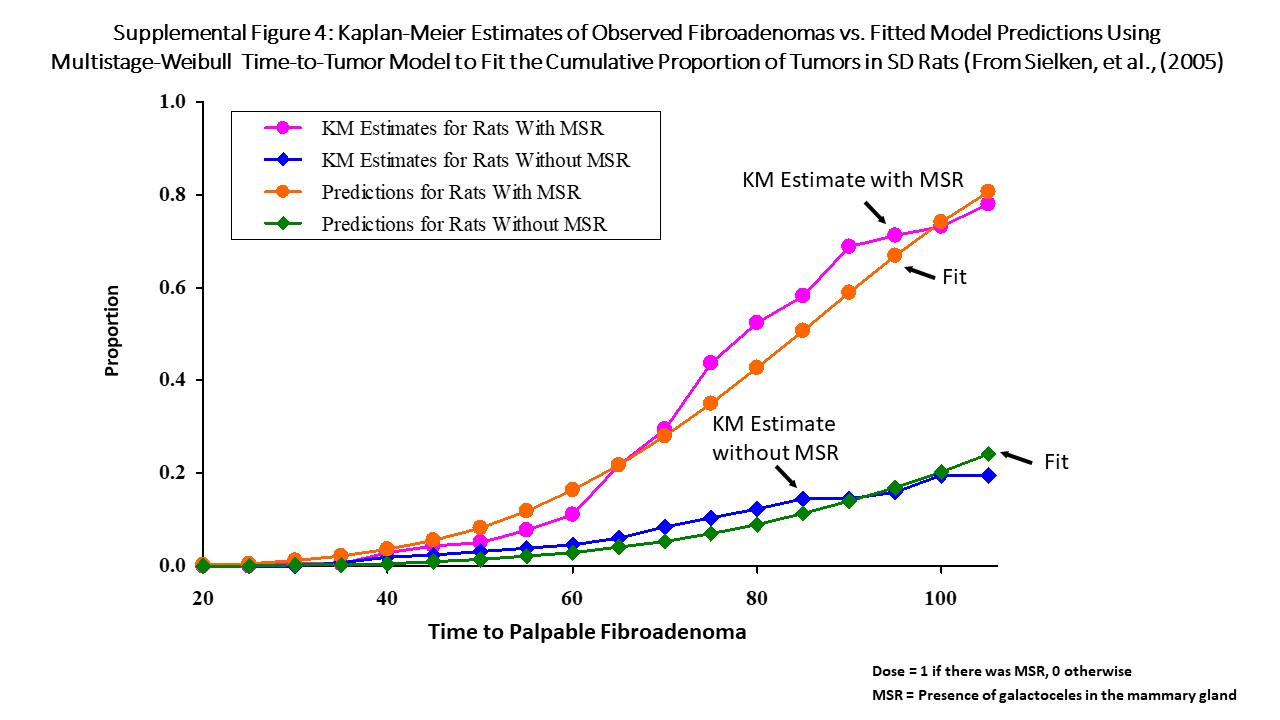

Supplement: Supplementary file 4 [file Image4.jpeg]

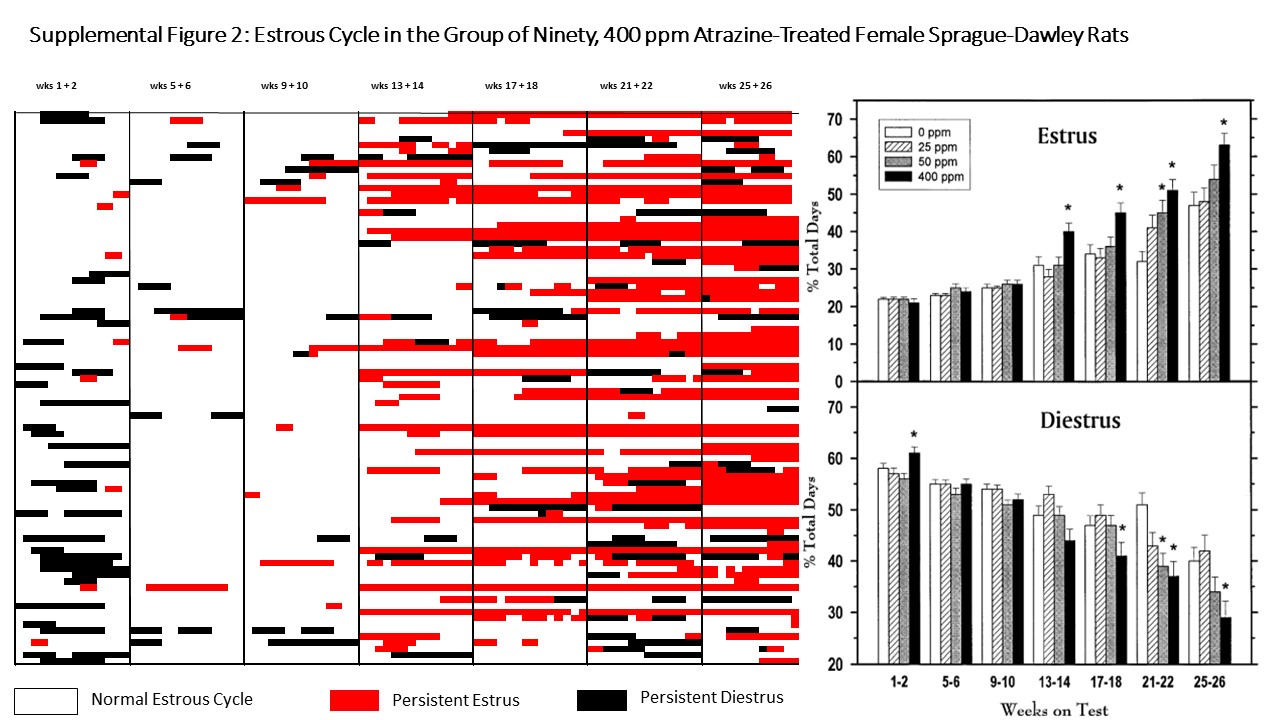

Supplement: Supplementary file 5 [file Image2.jpeg]

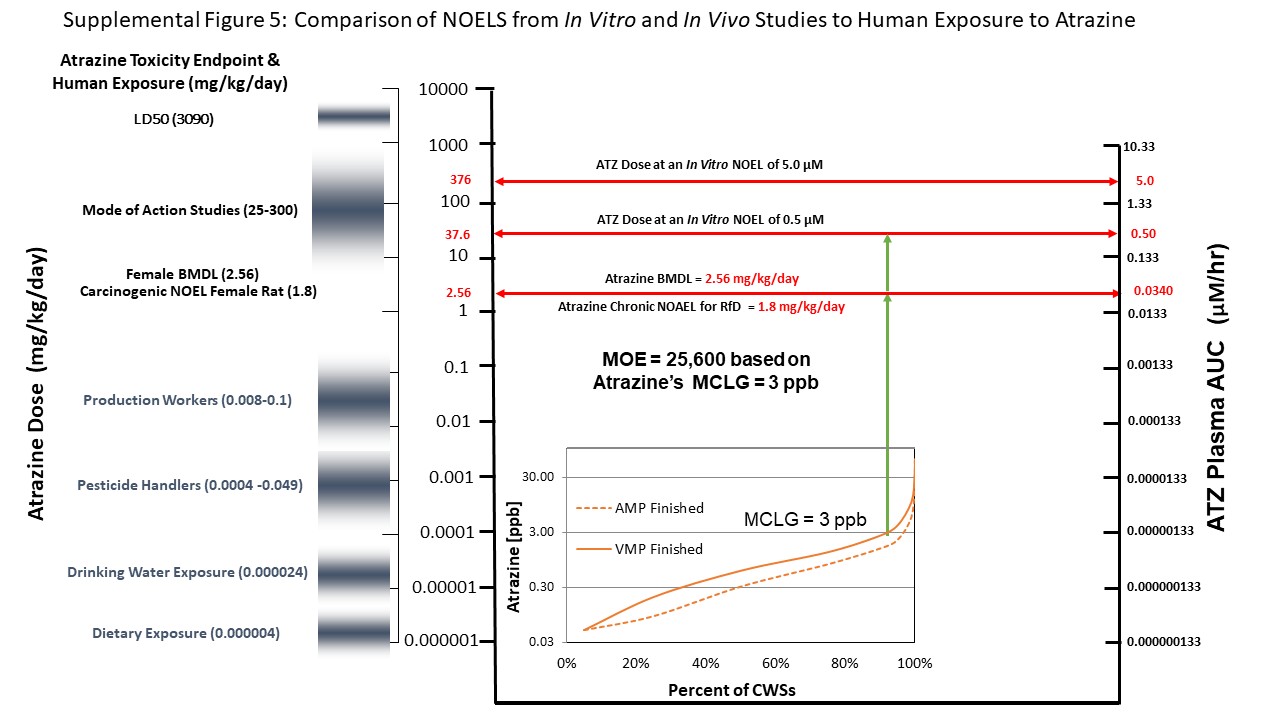

Supplement: Supplementary file 6 [file Image5.jpeg]
