# Supplementary material for: Effects of atrazine on the HPG and HPA axes and steroidogenic pathways in females: relevance to reproductive function and breast, ovarian and uterine cancer
Source: Front Toxicol. 2026 Jan 5;7:1686703. doi: 10.3389/ftox.2025.1686703 (PMC12812897; doi:10.3389/ftox.2025.1686703)
Supplement: Supplementary file 2 [file Supplementaryfile1.docx]

**Supplemental Figure Legends**

**Supplemental Figure 1:** **Estrous cycle in untreated female Sprague-Dawley rats (Eldridge et al., 1999b)**

The left panel displays the estrous cycle as 60 rows of color-coded, untreated female Sprague-Dawley rats for 2-week blocks of time, commencing on weeks 1 & 2 and ending on weeks 25 & 26. The animals were 6-7 weeks of age at the beginning of the observation period. During each 2-week block, daily vaginal smears were collected, evaluated, and classified as being either diestrus or proestrous smears based on vaginal cytology. The animal was considered to have a normal estrous cycle if the animal displayed 2 days of diestrus followed by a day of proestrus and up to 2 days of estrus, as illustrated in Figure 2. Regular cycles were displayed as white blocks of data. Persistent diestrus was defined by the presence of 3 or more successive days of diestrus smears (black blocks of data), and persistent estrous was defined as three or more successive days of estrus smears (red blocks of data).

The right panel presents the group mean (± SEM) and the percentage of total days spent in either estrus (top panel) or diestrus (bottom panel) for untreated (0 ppm) and atrazine-treated (25, 50, or 400 ppm) groups during the 26-week treatment period.

**Supplemental Figure 2**: **Estrous cycle in atrazine-treated female Sprague-Dawley rats (Eldridge, 1999b).**

The legend for Supplemental Figure 2 is the same as for Supplemental Figure 1.

**Supplemental Figure 3: Likelihoods for 34 different dose metrics used to characterize the adenocarcinoma outcomes relative to the likelihood of the outcome when the atrazine dose was used as the predictor variable (From Sielken et al., 2005).**

In the studies reported by Eldridge et al. (1999a, b), the likelihood (i.e., probability) of the occurrence of a mammary gland adenocarcinoma in an individual animal was estimated using a multistage-Weibull time-to-tumor model. The background likelihood was estimated for each animal, irrespective of dose group. The likelihood of expressing adenocarcinoma was estimated for each dose group, with the dose group serving as the predictor variable that best explained the individual animal’s likelihood of expressing an adenocarcinoma. Thirty-four other potential predictor variables were evaluated to determine if they increased (i.e., fold change in likelihood) the probability of predicting the individual animal’s probability of having the tumor. Among the 34 predictor variables assessed, the presence of an abnormal increase in the number of days in estrus resulted in the largest fold increase in correctly predicting the outcome (i.e., increased expression of adenocarcinomas).

**Supplemental Figure 4:** Kaplan-Meier Estimates of Observed Fibroadenomas vs. Fitted Model Predictions Using a Multistage-Weibull Time-to-Tumor Model to Fit the Cumulative Proportion of Tumors in SD Rats (From Sielken et al., 2005). Having information on whether the animal had mammary gland galactoceles (i.e., Mammary gland secretory activity [MSA]) substantially increases the probability that the animal will develop a mammary gland fibroadenoma compared to animals that did not have MSA.

**Supplemental Figure 5: Comparison of NOELS from *In Vitro* and *In Vivo* Studies to Human Exposure to Atrazine**

MCLG = Mean Contaminant Level Guideline (USEPA)

AMP Finished = Atrazine Community Water System (CWS) compliance monitoring data collected by States under the Safe Drinking Water Act (USEPA, 2011); Data from Tierney et al., 2008; Breckenridge et al., 2016)

VMP = Syngenta’s Voluntary Monitoring Program for vulnerable CWSs

AUC = Atrazine’s plasma area under the curve estimate (Data from Experiment 4, Simpkins et al., 2025).

Atrazine Benchmark Dose Lower Bound Estimate (BMDL). The BMDL of 2.56 mg/kg/day for atrazine was calculated by USEPA (2011b) based upon data collected by Cooper et al. (2010).

Atrazine Reference Dose (RfD) = 0.018 mg/kg/day based on a No Observed Effect Level (NOEL) of 1.8 mg/kg/day for the suppression of the LH surge in female rats. An uncertainty factor (UF) of 1000 was used to extrapolate the NOEL to humans.

The conversion of µM concentrations of atrazine in vitro to Area under the Curve (AUC) concentrations in vivo, and the oral gavage dose needed to produce the specific AUC, was based on the following regression equation developed by Simpkins et al. (2025).

Y = mX + b,

where Y = AUC (µM/L - hr); m = 0.0133 (Slope of linear trendline);

X = oral gavage dose (mg/kg/day)

b = 0 (Y intercept)

The Tier III estimated dietary dose was calculated by Bray (2008) for the US population based upon the reference dose (RfD) for atrazine and its chlorotriazine residues of 0.018 mg/kg/day. Tier III daily dietary exposure to atrazine and its chlorotriazine metabolites was determined to be 0.22% of the RfD.

The drinking water dose estimate at the MCLG of 3 ppb (3 µM/L) was calculated for a 60-kg person drinking 2 liters of water/day.

The pesticide handler exposure estimate was based on an evaluation provided by Lunchick and Selman (1998). They reported the lifetime average daily dose (LADD) for atrazine ranged from 0.008 to 0.1 mg/kg/day depending on the crop treated, method of application, job type, and whether open or closed systems and tractor cabs were used.

The estimated daily dose of atrazine for production workers was based on an estimate derived from urine monitoring data from Syngenta’s production facility in St. Gabriel, Louisiana (Baranyai, 1994). The range of doses estimated by Baranyai (1994) for production workers was comparable to those measured by Scutaru et al. (2002) in the plasma of workers exposed to atrazine in a Romanian production facility.

**References Cited in Supplemental Figures 1 to 5**

Baranyai, J.M. (1994). Triazine urine monitoring. Unpublished Technical Report, Ciba Crop Protection, Ciba-Geigy Corporation, PO Box 18300, Greensboro, NC 27419.

Bray, L. D., Szarka, A. Z., Heard, N. E., Hackett, D. S., & Kahrs, R. A. (2008). Dietary exposure assessment of the triazine herbicides. In H. M. LeBaron, J. E. McFarland, & O. C. Burnside (Eds.), *The Triazine Herbicides: 50 Years Revolutionizing Agriculture* (pp. 413-423). San Diego: Elsevier.

[Breckenridge CB, Campbell JL, Clewell HJ, Andersen ME, Valdez-Flores C, Sielken RL Jr. 2016. PBPK-Based Probabilistic Risk Assessment for Total Chlorotriazines in Drinking Water.](https://pubmed.ncbi.nlm.nih.gov/26794141/) *Toxicol Sci.*150:269-82.

Eldridge, J. C., Wetzel, L. T., & Tyrey, L. (1999b). Estrous cycle patterns of Sprague-Dawley rats during acute and chronic atrazine administration. *Reprod Toxicol, 13*(6), 491-499.

Eldridge, J. C., Wetzel, L. T., & Tyrey, L. (1999b). Estrous cycle patterns of Sprague-Dawley rats during acute and chronic atrazine administration. *Reprod Toxicol, 13*(6), 491-499.

Lunchick, C., & Selman, F. (1998). The Assessment of Worker Exposure to Atrazine and Simazine: A Tiered Approach. In *Triazine Herbicides: Risk Assessment* (pp. 141-157): American Chemical Society.

Sielken, R. L., Jr., Valdez-Flores, C., Holden, L. R., Breckenridge, C., & Stevens, J. (2005). Statistical inferences about the mechanism of action in carcinogenicity studies. *Scand J Work Environ Health, 31 Suppl 1*, 151-155; discussion 119-122.

Scutaru, B., Cozmei, C., Cazuc, V., Popa, D. and Hock, B. (2002). Immunoenzymatic analysis of atrazine exposure among manufacturing workers. J. Prev. Med. 19 (3): 31-36.

Tierney, D. P., Christensen, B. R., Dando, C., & Marut, K. M. (2008). Atrazine and simazine monitoring data in community water systems in the United States during 1993 to 2000. In H. M. LeBaron, J. E. McFarland, & O. C. Burnside (Eds.), *The Triazine Herbicides: 50 Years Revolutionizing Agriculture* (pp. 439-449). San Diego: Elsevier.

USEPA (2011). Re-evaluation of Human Health Effects of Atrazine: Review of Non-Cancer Effects, Drinking Water Monitoring Frequency, and Cancer Epidemiology, July 26–28, 2011, FIFRA Scientific Advisory Panel Meeting, p. 114. FIFRA Scientific Advisory Panel, Office of Science Coordination and Policy, United States Environmental Protection Agency, Arlington, VA.
