# Supplementary material for: Effects of atrazine on the HPG and HPA axes and steroidogenic pathways in females: relevance to reproductive function and breast, ovarian and uterine cancer
Source: Front Toxicol. 2026 Jan 5;7:1686703. doi: 10.3389/ftox.2025.1686703 (PMC12812897; doi:10.3389/ftox.2025.1686703)
Supplement: Supplementary file 7 [file Supplementaryfile2.docx]

List of Abbreviations

ACTH, adrenocorticotropic hormone

AKT, protein kinase B

AUC, area under the curve

AVP, arginine vasopressin

PKB, protein kinase B

AVPN, anteroventral periventricular nucleus

AVP, arginine vasopressin

CRH, corticotropin-releasing hormone

CNS, central nervous system

cAMP, cyclic adenosine monophosphate

CREB, cAMP response element-binding protein

DACT, diamino-chlorotriazine

DEA, deethylatrazine

DIA, deisopropylatrazine

EGFR, epidermal growth factor receptor

E2, 17β-estradiol

ER, estrogen receptor

ERE, estrogen-responsive elements,

F-344, Fischer-344

FSH, follicle-stimulating hormone

FMP, final menstrual period

GnRH, gonadotropin-releasing hormone

GPER, G-protein coupled estrogen receptor

GPR30, G-protein coupled estrogen receptor 30

cGMP, cyclic guanosine monophosphates

HPA, hypothalamic-pituitary-adrenal

HPG, hypothalamic-pituitary-gonadal

HPO, hypothalamic-pituitary-ovarian

HPT, hypothalamic-pituitary thyroid

KNDy, kisspeptin, neurokinin B, and dynorphin containing

Kiss1R, kisspeptin 1 receptor

LE, Long-Evans

LH, luteinizing hormone

LHR, luteinizing hormone receptor

MAPK, mitogen-activated protein kinases

MOA, modes of action

NOEL, no-observed-adverse-effect level

NOAEL, no-observed-adverse-effect level

PDE, phosphodiesterase

PI3K, phosphatidylinositol 3-kinases

PKA, protein kinase A

PLC, phospholipase C

PMD, Post-natal day

POA, preoptic nucleus

PR, progesterone receptor

RP3V, rostral periventricular areas of the third ventricle

SD, Sprague Dawley

SAR, structure-activity relationship

sc, subcutaneous

SCN, suprachiasmatic nucleus

SiRNA, small interfering RNA

shGPER, short hairpin RNA for GPER

steroidogenic factor 1 (SF-1)

TRH, thyrotropin-releasing hormone

VO, vaginal opening

VIP, vasoactive intestinal peptide
