# Supplementary material for: Effects of atrazine on the HPG and HPA axes and steroidogenic pathways in females: relevance to reproductive function and breast, ovarian and uterine cancer
Source: Front Toxicol. 2026 Jan 5;7:1686703. doi: 10.3389/ftox.2025.1686703 (PMC12812897; doi:10.3389/ftox.2025.1686703)
Supplement: Supplementary file 8 [file DataSheet1.docx]

**Supplemental Table 1: Effect of Atrazine on the Incidence of Mammary Tumors in Rodents (From Stevens et al. 1999)**


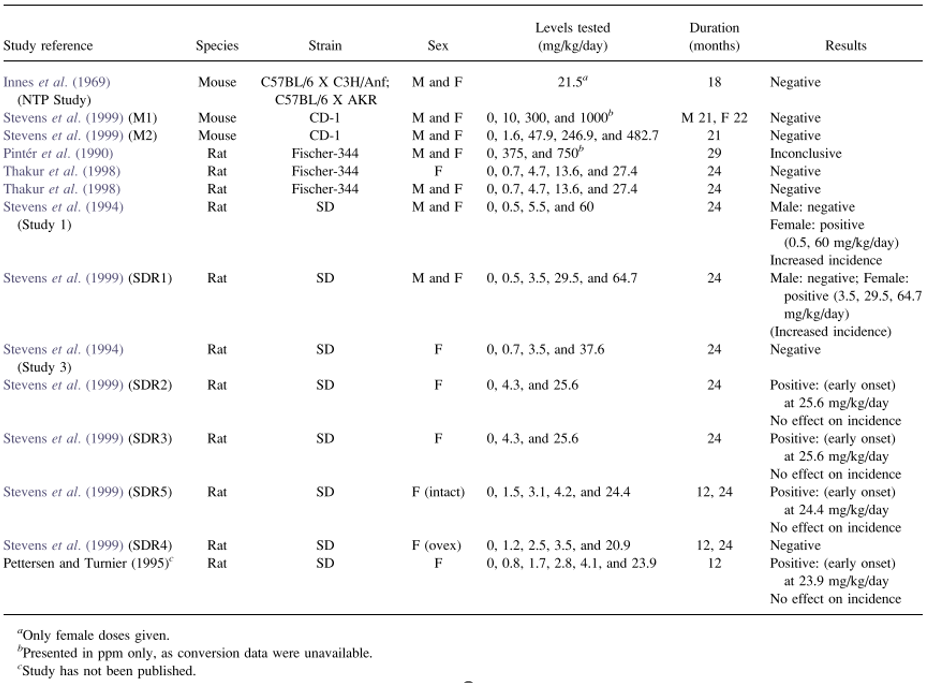


**Supplemental Table 2: Comparison of Reproductive Aging in Rats to Women (From Simpkins et al., 2011)**

| Parameter | SD Rat | Fishcer-344 Rat | Women |
| --- | --- | --- | --- |
| - Start of Senescence (% of normal lifespan) | 30-40 % | 60-70 % | 60-70% |
| - Principal cause of senescence | Hypothalamic failure to stimulate LH/FSH | Hypothalamic failure to control prolactin surges | Depletion of ovarian follicle content |
| - LH surge capability | Lost | Maintained | Maintained |
| - Predominant cycle pattern | Persistent estrus | Pseudopregnancy episodes | Menopause |
| - Estrogen/progesterone ratio | Elevated/prolonged | Reduced | Reduced |
| - Spontaneous mammary tumor incidence (lifetime) | 30-40 % | 2-5 % | 8-10 % |
| - Principal known factors that increase cancer risk | Prolactin, estrogen. | Prolactin, estrogen | Family history, parity, mutations, Adiposity |
| - Prolactin dependence | High | Medium | None |

**Supplemental Table 3. Studies that Evaluated Atrazine’s Effect on Estrogen Expression *In Vitro*^1^**

| Cell System | Tissue | Stimulated | Inhibited | Reference |
| --- | --- | --- | --- | --- |
| MCF-7 | ER-mediated proliferation | No | No | Connor (1996) |
| MCF-7 | ER-mediated proliferation | No | No | Soto (1995) |
| MCF-7 | ER-mediated proliferation | No | No | Fukamachi (2004) |
| MCF-7 | Nuclear DNA-PgR complex | No | No | Connor (1996) |
| MCF-7 | ER-mediated genetic expression | No | No | Connor (1996) |
| MCF-7 | ER-mediated genetic expression | No | No | Balaguer (1996) |
| HeLa | ER-mediated genetic expression | No | - | Balaguer (1996) |
| HeLa | Erα, ERꞵ-mediated expression | No | - | Balaguer (1996) |
| CHO cells | Erα, ERꞵ-mediated expression | No | - | Kojima (2004) |
| T47DLuc | ER-mediated genetic expression | No | - | Legler (2002) |
| MtT/E-2 | ER-mediated genetic expression | No | - | Fujimoto & Honda (2003) |
| Yeast | ER-mediated proliferation | No | Weak | Connor (1996) |
| Yeast | ER-mediated genetic expression | No | No | Tran (1996) |
| Yeast | ER-mediated genetic expression | No | - | Graumann (1999) |
| RTG-2 | AhR-expression | No | - | De la Casa-Resino (2014) |
| HEK-293 | Sea bass ERα expression | No | - | De la Casa-Resino (2014) |
| PD-DR-LUC | Avian thyroid receptor expression | No | - | De la Casa-Resino (2014) |
| Yeast | ER-mediated genetic expression | No | - | O’Connor (2000) |
| Clam gills | Metabolic stimulation | No | No | Cheney (1997) |
| Fish hepatic | Vitellogenin production | No | No/weak | Sanderson (2001) |
| Trout ER | Binding affinity | No | - | Shyu (2011) |
| Alligator/Human | Binding affinity | NB | NB | Rider (2010) |
| BG1 | Luciferase reporter ERα transactivation | No | - | Albanito (2015) |
| MCF7 | Luciferase reporter ERα transactivation | No | - | Albanito (2015) |
| Ishikawa | Luciferase reporter ERα transactivation | No | - | Albanito (2015) |
| SkBr3 | Luciferase reporter ERα transactivation | No | - | Albanito (2015) |
| SkBr3 | E2 binding to GPER &  ERK ½ phosphorylation | EC_20_ 10^-7^ M | - | Albanito (2015) |

1. Updated version of Table 2, Eldridge et al. (2008)

No = No response to atrazine at concentrations that were 10^5^-fold greater than the effective estradiol concentration.

NB = No Binding

Weak = Weak response to atrazine at concentrations that were 10^5^-fold greater than the effective estradiol concentration.

**Supplemental Table 4:** **Studies that Evaluated Atrazine’s Effect on Estrogen Expression *In Vivo***

| Species, Condition | Organ, Tissue, or Biological Endpoint | Stimulated | Inhibited | Reference |
| --- | --- | --- | --- | --- |
| Rat, OVX | Uterine weight | No | Weak | Tennant et al., 1994b |
| Rat, OVX | Uterine weight: Immature | No | Weak | Conner et al., 1996 |
| Rat, Intact | Uterine weight: Prepubertal | No | -- | Ashby et al. 2002 |
| Rat, Intact | Uterine weight: Prepubertal | No | Weak | Yamasaki et al. 2000 |
| Rat, OVX | Uterine thymidine incorporation | No | Weak | Tennant et al., 1994b |
| Rat, OVX | Uterine peroxidase reaction | No | Weak | Conner et al., 1996, 1998 |
|  |  |  |  |  |
| Rat, OVX | Progesterone receptor expression | No | Weak | Tennant et al., 1994b |
| Rat, OVX | Progesterone receptor expression | No | Weak | Conner et al., 1996 |
| AVPV, MPOA^1^ | Progesterone receptor mRNA | No | Weak | McMullin et al., 2004 |
|  |  |  |  |  |
| Rat, OVX | Mammary Tumor Incidence | No effect | -- | Stevens et al., 1999 |
| Rat, OVX | Transplanted pituitary tumor | No | -- | Fujimoto and Honda 2003 |
| Rat, Intact | DMBA-induced tumor growth | No | Inconclusive | Tanaka et al., 2004 |
|  |  |  |  |  |
| Rat, Intact | Reproductive tract development | No | -- | Eldridge et al., 1998 |
| Rat Intact | Reproductive tract development | No | Weak | Laws et al., 2000 |
| Rat Intact | Reproductive tract development | No | Weak | Laws et al., 2003 |
| Rat, Intact | Vaginal cytology cornification | No | -- | Eldridge et al., 1999b |
|  |  |  |  |  |
| Rat, OVX | Estrogen-primed LH surge | -- | Weak | Cooper et al., 2000 |
| Rat, OVX | Estrogen-primed LH surge | -- | Weak | Eldridge et al., 1999a |
| Rat, OVX | Estrogen-primed LH surge | -- | Weak | Simpkins et al., 1998 |
| Rat, OVX | Estrogen-primed Prolactin surge | -- | moderate | Cooper et al., 2000 |
| Rat, OVX | Estrogen-primed LH surge | Moderate increase/ decrease^2^ | | Goldman et al., 2013 |
| Rat, OVX | E2 + P4-primed LH surge | -- | 1 log unit | McMullin et al., 2004 |
|  |  |  |  |  |
| Alligator | Eggs, female phenotype | No | -- | Spiteiri et al., 2004 |
| Crocodile | Eggs, female phenotype | No | -- | Beldomenico et al.,2007 |
| Turtle | Feminization of testicular tissue | No | -- | DeSolla et al., 2006 |
| Frog | Female sex ratio of tadpoles | No | -- | Carr et al., 2003 |
| Goldfish | Vitellogenin production | No | -- | Spano et al., 2004 |
| Carp | Vitellogenin production | No | No effect | Sanderson et al., 2001 |
| Zebrafish | Vitellogenin mRNA | No | -- | Muncke et al., 2007 |
| Minnow | Female reproductive tissues | No | -- | Bringolf et al., 2004 |
| Japanese quail | Female oviduct weight, serum LH | No | -- | Wilhelms et al., 2006 |
| Japanese quail | Feminization: male reproductive tract | No | No effect | Wilhelms et al., 2006 |
| Fruit flies | Yolk protein genes | No | No effect | LeGoff et al., 2006 |

^1^ In situ hybridization of the progesterone receptor (PR) in the hypothalamus

AVPV = Anteroventral Periventricular Nucleus; MPOA = Medial Preoptic Area

^2^ Increased amplitude of the LH surge after a single dose, followed by decreased LH surges after 2 or 4 daily doses.

**Supplemental Table 5a:** **Epidemiological Studies on Breast Cancer**

| Study | Study Type | Included in  Sathiakumar (2011) | Included in  Boffetta (2013) | | Included in Meta-analysis |
| --- | --- | --- | --- | --- | --- |
| Epidemiological Studies on the Association Between Atrazine Exposure and Breast Cancer | | | | | |
| Kettles (1997) | Ecologic/Incidence Study of Triazine Use on Farms in KY | Yes | Yes | Yes | |
| MacLennan (2002) | Cancer Incidence: Production Facility Cohort, LA | Yes | Yes | Yes | |
| Hopenhyan-Rich (2002) | Ecologic/Incidence Study of Triazine Use on Farms in KY | Yes | Yes | Yes | |
| MacLennan (2003) | Mortality: Production Facility Cohort, LA | Yes | Yes | No^1^ | |
| Muir (2004) | Ecologic/Incidence Study of Atrazine in Two UK Counties | Yes | Yes | No^2^ | |
| Reynolds (2004) | Cancer Incidence – Ecologic Use Proximity, CA | Yes | No | No^3^ | |
| Reynolds (2005) | Cancer Incidence – Ecologic Use Proximity, CA | Yes | No | Yes | |
| Engel (2005) | AHS Cohort: Cancer Incidence | Yes | Yes | No^4^ | |
| Mills (2006) | Cancer Incidence – Ecologic Use: (lbs/county), CA | Yes | Yes | Yes | |
| McElroy (2007) | Cancer Incidence – Ecologic Exposure: (Well Water), WI | Yes | Yes | Yes | |
| Beane Freeman (2011) | AHS Prospective Cancer Incidence Study | No^5^ | Yes | No^4^ | |
| Werder (2020) | AHS Prospective Cancer Incidence Study | No^5^ | No^5^ | No^4^ | |
| Remigio (2024) | AHS Prospective Cancer Incidence Study | No^5^ | No^5^ | Yes | |

^1^ Excluded because MacLennan (2003) was a Cohort follow-up morality study.

^2^ Excluded because Muir (2004) used regression and autocorrelation analysis to evaluate breast cancer incidence clusters.

^3^Excluded because Reynolds (2004) was updated by Reynolds (2005);

^4^ Excluded these AHS cohort studies were updated by Remigio (2024)**;** ^5^ Study published after the review date.

**Supplemental Table 5b:** **Meta-Analysis of Epidemiological Studies on Breast Cancer**

**Supplemental Table 6a:** **Epidemiological Studies on Ovarian Cancer**

| Study | Study Type | Cited by Sathiakumar (2011) | Cited by Boffetta (2013) | Included in Meta-analysis |
| --- | --- | --- | --- | --- |
| Donna (1984) | Ecologic/Incidence Study of Herbicide Use on Farms in KY | Yes | No | No^1^ |
| Donna (1999) | Ecologic/Incidence Study of Triazine Use on Farms in KY | Yes | Yes | Yes |
| Van Leeuwen (1999) | Ecologic Exposure to Atrazine in Drinking Water: Null | Yes | No | No^2^ |
| Hopenhyan-Rich (2002) | Ecologic/Incidence Study of Triazine Use on Farms in KY | Yes | Yes | Yes |
| Young (2005) | Cancer Incidence: Ecologic Use on Farm in CA | Yes | Yes | Yes |
| Koutos (2010) | Cancer Incidence: AHS Cohort: Private Women Applicator | No | Yes | Yes ^3^ |
| Koutos (2010) | Cancer Incidence: AHS Cohort: Wives of Applicator | No | Yes | Yes ^3^ |
| Inoue-Choi (2016) | Cancer Incidence: IA Cohort: Drinking Water Exposure | No | No | Yes |
| Renier (2024) | Cancer Incidence: Agrican Cohort: Indirect Exposure | No | No | Yes |

1. Excluded because Donna (1984) was on herbicide use. Donna (1989) reported triazine use.
2. Excluded because Van Leeuwen (1999) did not find a correlation between exposure to atrazine in drinking water ovarian cancer incidence.
3. No data available for atrazine or the triazines; 70% of applicators used the triazines and 40% of the wives of applicators applied pesticides (Boffetta, 2013)

**Supplemental Table 6b:** **Meta-Analysis of the Associaton Between Atrazine Exposure and the Risk of Ovarian Cancer**

**References Cited in Supplemental Tables 1 to 4**

Albanito, L., Lappano, R., Madeo, A., Chimento, A., Prossnitz, E. R., Cappello, A. R., Maggiolini, M. (2015). Effects of atrazine on estrogen receptor alpha- and G protein-coupled receptor 30-mediated signaling and proliferation in cancer cells and cancer-associated fibroblasts. *Environ Health Perspect, 123*(5), 493-499.

Ashby, J., Tinwell, H., Stevens, J., Pastoor, T., & Breckenridge, C. B. (2002). The effects of atrazine on the sexual maturation of female rats. *Regul Toxicol Pharmacol, 35*(3), 468-473.

Balaguer, P., Joyeux, A., Denison, M. S., Vincent, R., Gillesby, B. E., & Zacharewski, T. (1996). Assessing the estrogenic and dioxin-like activities of chemicals and complex mixtures using *in vitro* recombinant receptor-reporter gene assays. *Can J Physiol Pharmacol, 74*(2), 216-222.

Beldomenico, P. M., Rey, F., Prado, W. S., Villarreal, J. C., Munoz-de-Toro, M., & Luque, E. H. (2007). In ovum exposure to pesticides increases the egg weight loss and decreases hatchlings weight of Caiman latirostris (Crocodylia: Alligatoridae). *EcoToxicol Environ Saf, 68*(2), 246-251.

Bringolf, R. B., Belden, J. B., & Summerfelt, R. C. (2004). Effects of atrazine on fathead minnow in a short-term reproduction assay. *Environ Toxicol Chem, 23*(4), 1019-1025.

Carr, J. A., Gentles, A., Smith, E. E., Goleman, W. L., Urquidi, L. J., Thuett, K., . . . Van Der Kraak, G. (2003). Response of larval Xenopus laevis to atrazine: assessment of growth, metamorphosis, and gonadal and laryngeal morphology. *Environ Toxicol Chem, 22*(2), 396-405.

Cheney, M. A., Fiorillo, R., & Criddle, R. S. (1997). Herbicide and estrogen effects on the metabolic activity of Elliptio complanata measured by calorespirometry. *Comp Biochem Physiol C Pharmacol Toxicol Endocrinol, 118*(2), 159-164.

Connor, K., Howell, J., Chen, I., Liu, H., Berhane, K., Sciarretta, C., Zacharewski, T. (1996). Failure of chloro-S-triazine-derived compounds to induce estrogen receptor-mediated responses *in vivo* and *in vitro*. *Fundam Appl Toxicol, 30*(1), 93-101.

Connor, K., Howell, J., Safe, S., Chen, I., Liu, H., Berhane, K., Zacharewski, T. (1998). Failure of Chloro-s-triazine-derived compounds to induce estrogenic responses *in vivo* and *in vitro*. In *Triazine Herbicides: Risk Assessment* (pp. 424-431): American Chemical Society.

Cooper, R. L., Stoker, T. E., Tyrey, L., Goldman, J. M., & McElroy, W. K. (2000). Atrazine disrupts the hypothalamic control of pituitary-ovarian function. *Toxicol Sci, 53*(2), 297-307.

De la Casa-Resino, I., Navas, J. M., & Fernandez-Cruz, M. L. (2014). Chlorotriazines do not activate the aryl hydrocarbon receptor, the oestrogen receptor or the thyroid receptor in *in vitro* assays. *Altern Lab Anim, 42*(1), 25-30.

de Solla, S. R., Martin, P. A., Fernie, K. J., Park, B. J., & Mayne, G. (2006). Effects of environmentally relevant concentrations of atrazine on gonadal development of snapping turtles (Chelydra serpentina). *Environ Toxicol Chem, 25*(2), 520-526.

Eldridge, J. C., Wetzel, L. T., Stevens, J. T., & Simpkins, J. W. (1999a). The mammary tumor response in triazine-treated female rats: a threshold-mediated interaction with strain and species-specific reproductive senescence. *Steroids, 64*(9), 672-678.

Eldridge, J. C., Wetzel, L. T., & Tyrey, L. (1999b). Estrous cycle patterns of Sprague-Dawley rats during acute and chronic atrazine administration. *Reprod Toxicol, 13*(6), 491-499.

Eldridge, J. C., Stevens, J. T., & Breckenridge, C. B. (2008). Atrazine interaction with estrogen expression systems. *Rev Environ Contam Toxicol, 196*, 147-160.

Fujimoto, N. & Honda, H. (2003). Effects of environmental estrogenic compounds on growth of a transplanted estrogen responsive pituitary tumor cell line in rats. *Food Chem Toxicol, 41*(12), 1711-1717.

Fukamachi, K., Han, B. S., Kim, C. K., Takasuka, N., Matsuoka, Y., Matsuda, E., Tsuda, H. (2004). Possible enhancing effects of atrazine and nonylphenol on 7,12-dimethylbenz[a]anthracene-induced mammary tumor development in human c-Ha-ras proto-oncogene transgenic rats. *Cancer Sci, 95*(5), 404-410.

Goldman, J., Davis, L. K., Murr, A. S., & Cooper, R. L. (2013). Atrazine-induced elevation or attenuation of the LH surge in the ovariectomized, estrogen-primed female rat: role of adrenal progesterone. *Reproduction, 146*(4), 305-314.

Graumann, K., Breithofer, A., & Jungbauer, A. (1999). Monitoring of estrogen mimics by a recombinant yeast assay: synergy between natural and synthetic compounds? *Sci Total Environ, 225*(1-2), 69-79.

Kojima, H., Katsura, E., Takeuchi, S., Niiyama, K., & Kobayashi, K. (2004). Screening for estrogen and androgen receptor activities in 200 pesticides by *in vitro* reporter gene assays using Chinese hamster ovary cells. *Environ Health Perspect, 112*(5), 524-531.

Laws, S. C., Ferrell, J. M., Stoker, T. E., Schmid, J., & Cooper, R. L. (2000). The effects of atrazine on female wistar rats: an evaluation of the protocol for assessing pubertal development and thyroid function. *Toxicol Sci, 58*(2), 366-376.

Laws, S. C., Ferrell, J. M., Stoker, T. E., & Cooper, R. L. (2003). Pubertal development in female Wistar rats following exposure to propazine and atrazine biotransformation by-products, diamino-S-chlorotriazine and hydroxyatrazine. *Toxicol Sci, 76*(1), 190-200.

Legler, J., Dennekamp, M., Vethaak, A. D., Brouwer, A., Koeman, J. H., van der Burg, B., & Murk, A. J. (2002). Detection of estrogenic activity in sediment-associated compounds using in vitro reporter gene assays. *Sci Total Environ, 293*(1-3), 69-83. doi:10.1016/s0048-9697(01)01146-9.

Le Goff, G., Hilliou, F., Siegfried, B. D., Boundy, S., Wajnberg, E., Sofer, L., . . . Feyereisen, R. (2006). Xenobiotic response in Drosophila melanogaster: sex dependence of P450 and GST gene

McMullin, T. S., Andersen, M. E., Nagahara, A., Lund, T. D., Pak, T., Handa, R. J., & Hanneman, W. H. (2004). Evidence that atrazine and diaminochlorotriazine inhibit the estrogen/progesterone induced surge of luteinizing hormone in female Sprague-Dawley rats without changing estrogen receptor action. *Toxicol Sci, 79*(2), 278-286.

Muncke, J., Junghans, M., & Eggen, R. I. (2007). Testing estrogenicity of known and novel (xeno-)estrogens in the MolDarT using developing zebrafish (Danio rerio). *Environ Toxicol, 22*(2), 185-193.

O'Connor, J. C., Plowchalk, D. R., Van Pelt, C. S., Davis, L. G., & Cook, J. C. (2000). Role of prolactin in chloro-S-triazine rat mammary tumorigenesis. *Drug Chem Toxicol, 23*(4), 575-601.

Petterson, J.C. & Turnier (1995). One year chronic toxicity study on atrazine in Sprague-Dawley rats. Study Number F-0071, Ciba-Geigy Corporation, Greensboro, NC.

Rider, C. V., Hartig, P. C., Cardon, M. C., Lambright, C. R., Bobseine, K. L., Guillette, L. J., Jr., Wilson, V. S. (2010). Differences in sensitivity but not selectivity of xenoestrogen binding to alligator versus human estrogen receptor alpha. *Environ Toxicol Chem, 29*(9), 2064-2071.

Sanderson, J. T., Letcher, R. J., Heneweer, M., Giesy, J. P., & van den Berg, M. (2001). Effects of chloro-s-triazine herbicides and metabolites on aromatase activity in various human cell lines and on vitellogenin production in male carp hepatocytes. *Environ Health Perspect, 109*(10), 1027-1031.

Shyu, C., Cavileer, T. D., Nagler, J. J., & Ytreberg, F. M. (2011). Computational estimation of rainbow trout estrogen receptor binding affinities for environmental estrogens. *Toxicol Appl Pharmacol, 250*(3), 322-326.

Simpkins, J. W., Eldridge, J. C., & Wetzel, L. T. (1998). Role of Strain-Specific Reproductive Patterns in the Appearance of Mammmary Tumors in Atrazine-Treated Rats. In *Triazine Herbicides: Risk Assessment* (pp. 399-413): American Chemical Society.

Simpkins, J. W., Swenberg, J. S., Weiss, N., Brusick, D., Eldridge, J. C., Stevens, J. T., . . . Breckenridge, C. B. (2011). Atrazine and breast cancer: A framework assessment of the toxicological and epidemiological evidence. *Toxicol Sci, 123*(2), 441-459.

Soto, A. M., Sonnenschein, C., Chung, K. L., Fernandez, M. F., Olea, N., & Serrano, F. O. (1995). The E-SCREEN assay as a tool to identify estrogens: an update on estrogenic environmental pollutants. *Environ Health Perspect, 103 Suppl 7*(Suppl 7), 113-122.

Spano, L., Tyler, C. R., van Aerle, R., Devos, P., Mandiki, S. N., Silvestre, F., . . . Kestemont, P. (2004). Effects of atrazine on sex steroid dynamics, plasma vitellogenin concentration and gonad development in adult goldfish (Carassius auratus). *Aquat Toxicol, 66*(4), 369-379.

Spiteri, I. D., Guillette, L. J., Jr., & Crain, D. A. (1999). The functional and structural observations of the neonatal reproductive system of alligators exposed in ovo to atrazine, 2,4-D, or estradiol. *Toxicol Ind Health, 15*(1-2), 181-186.

Stevens, J. T., Breckenridge, C. B., Wetzel, L. T., Gillis, J. H., Luempert, L. G., III, & Eldridge, J. C. (1994). Hypothesis for mammary tumorigenesis in Sprague-Dawley rats exposed to certain triazine herbicides. *J Toxicol Environ Health, 43*(2), 139-153.

Stevens, J. T., Breckenridge, C. B., Wetzel, L., Thakur, A. K., Liu, C., Werner, C.,
Eldridge, J. C. (1999). A risk characterization for atrazine: oncogenicity profile. *J Toxicol Environ Health A, 56*(2), 69-109.

Tanaka, T., Kohno, H., Suzuki, R. and Sugie, S. . (2004). Lack of modifying effects of an estrogenic compound atrazine on 7,12-dimethylbenz(a anthracene-induced ovarian carcinogenesis in rats. *Cancer Lett, 2010*, 129-137.

Tennant, M. K., Hill, D. S., Eldridge, J. C., Wetzel, L. T., Breckenridge, C. B., & Stevens, J. T. (1994a). Chloro-s-triazine antagonism of estrogen action: limited interaction with estrogen receptor binding. *J Toxicol Environ Health, 43*(2), 197-211.

Tennant, M. K., Hill, D. S., Eldridge, J. C., Wetzel, L. T., Breckenridge, C. B., & Stevens, J. T. (1994b). Possible antiestrogenic properties of chloro-s-triazines in rat uterus. *J Toxicol Environ Health, 43*(2), 183-196.

Thakur, A. K., Wetzel, L. T., Voelker, R. W., & Wakefield, A. E. (1998). Results of a Two-Year Oncogenicity Study in Fischer 344 Rats with Atrazine. In *Triazine Herbicides: Risk Assessment* (pp. 384-398): American Chemical Society.

Tran, D. Q., Kow, K. Y., McLachlan, J. A., & Arnold, S. F. (1996). The inhibition of estrogen receptor-mediated responses by chloro-S-triazine-derived compounds is dependent on estradiol concentration in yeast. *Biochem Biophys Res Commun, 227*(1), 140-146.

Wilhelms, K. W., Cutler, S. A., Proudman, J. A., Anderson, L. L., & Scanes, C. G. (2005). Atrazine and the hypothalamo-pituitary-gonadal axis in sexually maturing precocial birds: studies in male Japanese quail. *Toxicol Sci, 86*(1), 152-160.

Wilhelms, K. W., Cutler, S. A., Proudman, J. A., Carsia, R. V., Anderson, L. L., & Scanes, C. G. (2006). Lack of effects of atrazine on estrogen-responsive organs and circulating hormone concentrations in sexually immature female Japanese quail (Coturnix coturnix japonica). *Chemosphere, 65*(4), 674-681.

Yamasaki, K., Sawaki, M., Noda, S., Muroi, T., & Maekawa, A. (2000). Immature Rat Uterotrophic Assay of Diethylstilbestrol, Ethynyl Estradiol and Atrazine. *J Toxicol Pathol, 13*(3), 145-149.

**References for Epidemiology Studies Cited in Supplemental Tables 5 & 6**

Beane Freeman L, Rusiecki JA, Hoppin JA, Lubin JH, Koutros S, Andreotti G, et al. (2011). Atrazine and cancer incidence among pesticide applicators in the Agricultural Health Study (1994–2007). Environ Health Persp 119:1253–1259.

Boffetta, P., Adami, H. O., Berry, S. C., & Mandel, J. S. (2013). Atrazine and cancer: a review of the epidemiologic evidence. *Eur J Cancer Prev, 22*(2), 169-180.

Donna, A., Betta, P. G., Robutti, F., Crosignani, P., Berrino, F., & Bellingeri, D. (1984). Ovarian mesothelial tumors and herbicides: a case-control study. *Carcinogenesis, 5*(7), 941-942.

Donna, A., Crosignani, P., Robutti, F., Betta, P. G., Bocca, R., Mariani, N., Berrino, F. (1989). Triazine herbicides and ovarian epithelial neoplasms. *Scand J Work Environ Health, 15*(1), 47-53.

Engel, L. S., Hill, D. A., Hoppin, J. A., Lubin, J. H., Lynch, C. F., Pierce, J., Alavanja, M. C. (2005). Pesticide use and breast cancer risk among farmers' wives in the agricultural health study. *Am J Epidemiol, 161*(2), 121-135.

Hopenhayn-Rich, C., Stump, M. L., & Browning, S. R. (2002). Regional assessment of atrazine exposure and incidence of breast and ovarian cancers in Kentucky. *Arch Environ Contam Toxicol, 42*(1), 127-136.

Inoue-Choi, M., Weyer, P. J., Jones, R. R., Booth, B. J., Cantor, K. P., Robien, K., & Ward, M. H. (2016). Atrazine in public water supplies and risk of ovarian cancer among postmenopausal women in the Iowa Women's Health Study. *Occup Environ Med, 73*(9), 582-587.

Kettles, M. K., Browning, S. R., Prince, T. S., & Horstman, S. W. (1997). Triazine herbicide exposure and breast cancer incidence: an ecologic study of Kentucky counties. *Environ Health Perspect, 105*(11), 1222-1227.

Koutros, S., Alavanja, M. C., Lubin, J. H., Sandler, D. P., Hoppin, J. A., Lynch, C. F.,
Freeman, L. E. (2010). An update of cancer incidence in the Agricultural Health Study. *J Occup Environ Med, 52*(11), 1098-1105.

MacLennan, P. A., Delzell, E., Sathiakumar, N., Myers, S. L., Cheng, H., Grizzle, W., Wu, X. C. (2002). Cancer incidence among triazine herbicide manufacturing workers. *J Occup Environ Med, 44*(11), 1048-1058.

MacLennan, P. A., Delzell, E., Sathiakumar, N., & Myers, S. L. (2003). Mortality among triazine herbicide manufacturing workers. *J Toxicol Environ Health A, 66*(6), 501-517.

McElroy, J. A., Gangnon, R. E., Newcomb, P. A., Kanarek, M. S., Anderson, H. A.,
Brook, J. V., Remington, P. L. (2007). Risk of breast cancer for women living in rural areas from adult exposure to atrazine from well water in Wisconsin. *J Expo Sci Environ Epidemiol, 17*(2), 207-214.

Mills, P. K., & Yang, R. (2006). Regression analysis of pesticide use and breast cancer incidence in California Latinas. *J Environ Health, 68*(6), 15-22; quiz 43-14.

Muir, K., Rattanamongkolgul, S., Smallman-Raynor, M., Thomas, M., Downer, S., & Jenkinson, C. (2004). Breast cancer incidence and its possible spatial association with pesticide application in two counties of England. *Public Health, 118*(7), 513-520.

Remigio, R. V., Andreotti, G., Sandler, D. P., Erickson, P. A., Koutros, S., Albert, P. S., Beane Freeman, L. E. (2024). An Updated Evaluation of Atrazine-Cancer Incidence Associations among Pesticide Applicators in the Agricultural Health Study Cohort. *Environ Health Perspect, 132*(2), 27010.

Renier, M., Hippert, J., Louis-Bastien, W., Tual, S., Meryet-Figuiere, M., Vigneron, N., group, A. (2024). Agricultural exposure and risk of ovarian cancer in the AGRIculture and CANcer (AGRICAN) cohort. *Occup Environ Med, 81*(2), 75-83.

Reynolds, P., Hurley, S. E., Goldberg, D. E., Yerabati, S., Gunier, R. B., Hertz, A., California Teachers, S. (2004). Residential proximity to agricultural pesticide use and incidence of breast cancer in the California Teachers Study cohort. *Environ Res, 96*(2), 206-218.

Reynolds, P., Hurley, S. E., Gunier, R. B., Yerabati, S., Quach, T., & Hertz, A. (2005). Residential proximity to agricultural pesticide use and incidence of breast cancer in California, 1988-1997. *Environ Health Perspect, 113*(8), 993-1000.

Sathiakumar, N., MacLennan, P. A., Mandel, J., & Delzell, E. (2011). A review of epidemiologic studies of triazine herbicides and cancer. *Crit Rev Toxicol, 41*(suppl 1), 1-34.

Van Leeuwen, J. A., Waltner-Toews, D., Abernathy, T., Smit, B., & Shoukri, M. (1999). Associations between stomach cancer incidence and drinking water contamination with atrazine and nitrate in Ontario (Canada) agroecosystems, 1987-1991. *Int J Epidemiol, 28*(5), 836-840.

Werder, E. J., Engel, L. S., Satagopan, J., Blair, A., Koutros, S., Lerro, C. C., . . . Beane Freeman, L. E. (2020). Herbicide, fumigant, and fungicide use and breast cancer risk among farmers' wives. *Environ Epidemiol, 4*(3), e097.

Young, H. A., Mills, P. K., Riordan, D. G., & Cress, R. D. (2005). Triazine herbicides and epithelial ovarian cancer risk in central California. *J Occup Environ Med, 47*(11), 1148-1156.
